# External exposome and incident asthma across the life course in 14 European cohorts: a prospective analysis within the EXPANSE project

**DOI:** 10.1016/j.lanepe.2025.101314

**Published:** 2025-05-15

**Authors:** Zhebin Yu, Sara Kress, Natalia Blay, Petr Gregor, Hanna-Maria Kukk, Miriam Leskien, Renata Majewska, Max J. Oosterwegel, Daniel Szabó, Margreet ten Have, Jana Klánová, Ondřej Mikeš, Anna Bergström, Alonso Bussalleu, Rafael de Cid, Andrea Dalecka, Payam Dadvand, Saskia van Dorsselaer, Krista Fischer, Kees de Hoogh, Gerard H. Koppelman, Jaanika Kronberg, Estonian Biobank Research Team, Andres Metspalu, Lili Milani, Tõnu Esko, Mait Metspalu, Jeroen Lakerveld, Petter Ljungman, Simon Kebede Merid, Pawel Macek, Marta Manczuk, Anne-Sophie Merritt, Agnieszka Pac, Priit Palta, Göran Pershagen, Annette Peters, Hynek Pikhart, Apolline Saucy, Tamara Schikowski, Youchen Shen, Marie Standl, Cathryn Tonne, Roel Vermeulen, Jelle Vlaanderen, Judith M. Vonk, Kathrin Wolf, Carl Henrik Ek, Olena Gruzieva, Ulrike Gehring, Erik Melén, EXPANSE project

**Affiliations:** a Institute of Environmental Medicine, Karolinska Institutet, Stockholm, Sweden; b IUF – Leibniz Research Institute for Environmental Medicine, Düsseldorf, Germany; c Genomes for Life-GCAT Lab, CORE Program, Germans Trias I Pujol Research Institute (IGTP), Badalona, Spain; d Grup de Recerca en Impacte de Les Malalties Cròniques I Les Seves Trajectòries (GRIMTra) (IGTP), Badalona, Spain; e RECETOX, Faculty of Science, Masaryk University, Brno, Czech Republic; f Institute of Genomics, University of Tartu, Estonia; g Institute of Mathematics and Statistics, University of Tartu, Estonia; h Institute of Epidemiology, Helmholtz Zentrum München - German Research Center for Environmental Health, Neuherberg, Germany; i Institute for Medical Information Processing, Biometry and Epidemiology, Medical Faculty, Ludwig-Maximilians-Universität München, Munich, Germany; j Chair of Epidemiology and Preventive Medicine, Jagiellonian University Medical College, Kraków, Poland; k Institute for Risk Assessment Sciences, Utrecht University, Utrecht, the Netherlands; l Netherlands Institute of Mental Health and Addiction, Utrecht, the Netherlands; m Centre for Occupational and Environmental Medicine, Region Stockholm, Stockholm, Sweden; n Swiss Tropical and Public Health Institute, Allschwil, Switzerland; o University of Basel, Basel, Switzerland; p ISGlobal, Barcelona, Spain; q Universitat Pompeu Fabra (UPF), Barcelona, Spain; r CIBER Epidemiología y Salud Pública, Spain; s University of Groningen, University Medical Center Groningen, Beatrix Children’s Hospital, Department of Pediatric Pulmonology and Pediatric Allergology, Groningen, the Netherlands; t University of Groningen, University Medical Center Groningen, Groningen Research Institute for Asthma and COPD (GRIAC), Groningen, the Netherlands; u Department of Epidemiology and Data Science, Amsterdam UMC, Location Vrije Universiteit Amsterdam, Amsterdam, the Netherlands; v Department of Cardiology, Danderyd Hospital, Stockholm, Sweden; w Department of Clinical Sciences and Education, Södersjukhuset, Karolinska Institutet, Stockholm, Sweden; x Collegium Medicum, Jan Kochanowski University of Kielce, Poland; y Scientific Research, Epidemiology and R&D Centre, Holycross Cancer Centre, Kielce, Poland; z Department of Cancer Epidemiology and Primary Prevention, Maria Sklodowska-Curie National Research Institute of Oncology, Warsaw, Poland; aa Chair of Epidemiology, Institute for Medical Information Processing, Biometry and Epidemiology, Médical Faculty, Ludwig-Maximilians-Universit at München, Munich, Germany; ab Munich Heart Alliance, German Center for Cardiovascular Health (DZHK e.V., partner-site Munich), Munich, Germany; ac Institute of Social and Preventive Medicine (ISPM), University of Berne, Berne, Switzerland; ad Department of Environment and Health, School of Public Health, University of Bielefeld, Bielefeld, Germany; ae German Center for Lung Research (DZL), Munich, Germany; af German Center for Child and Adolescent Health (DZKJ), Munich, Germany; ag Department of Epidemiology, University of Groningen, University Medical Center Groningen, Groningen, the Netherlands; ah Department of Computer Science and Technology, University of Cambridge, Cambridge, Cambridgeshire, United Kingdom

**Keywords:** Exposome, Asthma, Life course, Cohort

## Abstract

**Background:**

The joint impact of exposure to multiple urban environmental factors on asthma remains unclear.

**Methods:**

We analysed data from 14 European cohorts to assess the impact of the urban exposome on asthma incidence across the life course. We linked three external exposome domains (air pollution, built environment, ambient temperature) to the participants’ home addresses at baseline. We performed k-means clustering within each domain and assessed associations of clusters with asthma adjusting for potentially relevant covariates in cohort-specific analyses, with subsequent separate meta-analyses for birth and adult cohorts. An environmental risk score using a coefficient-weighted sum approach was used to assess the impact of combining the three domains.

**Findings:**

A total of 7428 incident asthma cases were identified among 349,037 participants (from birth up to age 70+). Overall, we observed higher risks of asthma for clusters characterized by high particulate matter and nitrogen dioxide exposure in adults (OR_meta_ = 1.13, 95%CI:1.01–1.25), and clusters characterized by high built-up area and low levels of greenness in both children and adults (OR_meta_ = 1.36, 95%CI: 1.14–1.64 for birth cohorts and OR_meta_ = 1.15, 95%CI: 1.03–1.28 for adult cohorts, respectively). The joint exposure using the environment risk score combining the three domains was consistently associated with higher risks of incident asthma (OR_meta_ = 1.13, 95%CI: 1.07–1.20 for birth cohorts, OR_meta_ = 1.15, 95%CI: 1.10–1.20 for adult cohorts per 20% increase). On average 11.6% of the incident asthma cases could be attributed to environmental risk score above cohort-specific median levels.

**Interpretation:**

Multiple environmental exposures jointly contribute to incident asthma risk across the life course. Urban planning accounting for these factors may help mitigate asthma development.

**Funding:**

This study was funded by the 10.13039/501100007601European Union’s Horizon 2020 research and innovation program under agreement No 874627 (EXPANSE).

**Research in context**

**Evidence before this study**

Several environmental exposures have been identified as risk factors for asthma onset. However, most evidence comes from single-exposure analyses, which fail to capture the complex, real-life patterns of multiple exposures. We searched PubMed and Web of Science for peer reviewed studies using the search term ((“Exposure” and “Environment”) AND (“Exposome”)) AND (“Asthma” and “Epidemiology”) from inception up to September 30th, 2024. Only three studies have reported the association between multiple environmental exposures and asthma using exposomic approaches. Two studies were limited to a single cohort with cross-sectional analysis, and one combined data from six cohorts; they varied in their exposure assessment methods, and focused solely on asthma-related prevalence outcomes, rather than asthma incidence.

**Added value of this study**

This study analysed data from 14 European cohorts with harmonized exposure assessments. We included asthma incidence data across the lifespan, from birth to old age, and leveraged this data for longitudinal analyses. To address the complexity of real-life exposure patterns, we applied clustering methods to evaluate the joint associations of multiple environmental exposures.

**Implications of all the available evidence**

Our finding provides evidence that multiple environmental exposures jointly contribute to incident asthma risk across the life course. Integrating these factors into urban planning and policy development may help mitigate asthma onset and promote healthier living environments.

## Introduction

Asthma is one of the most common chronic non-communicable diseases worldwide.^1^^,^^2^ Due to the complex etiology of asthma, a better understanding of potentially modifiable risk factors is desired to develop efficient public health primary and secondary preventive measures.^3^^,^^4^ Notably, asthma prevalence is higher in urban compared to rural settings. As 50% of the global population is currently living in urban settings and this is expected to increase up to 70% by 2050,^5^ it is essential to evaluate the impact of the urban environment on asthma.^6^^,^^7^ Several exposures related to the urban environment have been identified as risk factors for asthma, including traffic-related air pollution,^8^ environmental tobacco smoke^9^ and social deprivation.^10^ However, previous studies typically focused on single exposures, which do not reflect the complexity of multi-exposure patterns in real life.

To our knowledge, only three studies so far have investigated the joint impact of multiple environmental exposures and asthma-related outcomes from an exposome perspective.^11^, ^12^, ^13^ Of these, two studies were conducted among children and one among adults. These studies applied a clustering approach to identify subgroups with distinct multi-exposure profiles and linked these to asthma-related outcomes. However, each of these studies included different types of environmental exposure, making it challenging to compare the identified exposure profiles across studies. In addition, two studies were restricted to single cohorts and one study combined data from six existing cohorts. All of these studies assessed the asthma outcome at one single timepoints and none investigated incident asthma. Therefore, there is a need for further studies using harmonized analyses of multiple cohorts and longitudinal designs to evaluate the joint effect of multiple environmental exposures.

The aim of the current study was to investigate the association of both single and joint environmental exposures from three major domains (air pollution, built environment, ambient temperature) with incident asthma, i.e., new-onset asthma. As a secondary aim, we estimated the proportion of incident asthma cases that was attributable to joint exposures. We included mature birth cohorts (birth cohorts with follow-up examinations up to young adulthood) as well as adult cohorts. This study was conducted within the ‘EXposome Powered tools for healthy living in urbAN SEttings (EXPANSE)’ project, which focuses on investigating the complex mixture of the urban environment on cardio-metabolic and pulmonary diseases in Europe using an exposome approach.^14^

## Methods

### Study population and design

**Fig. 1:**
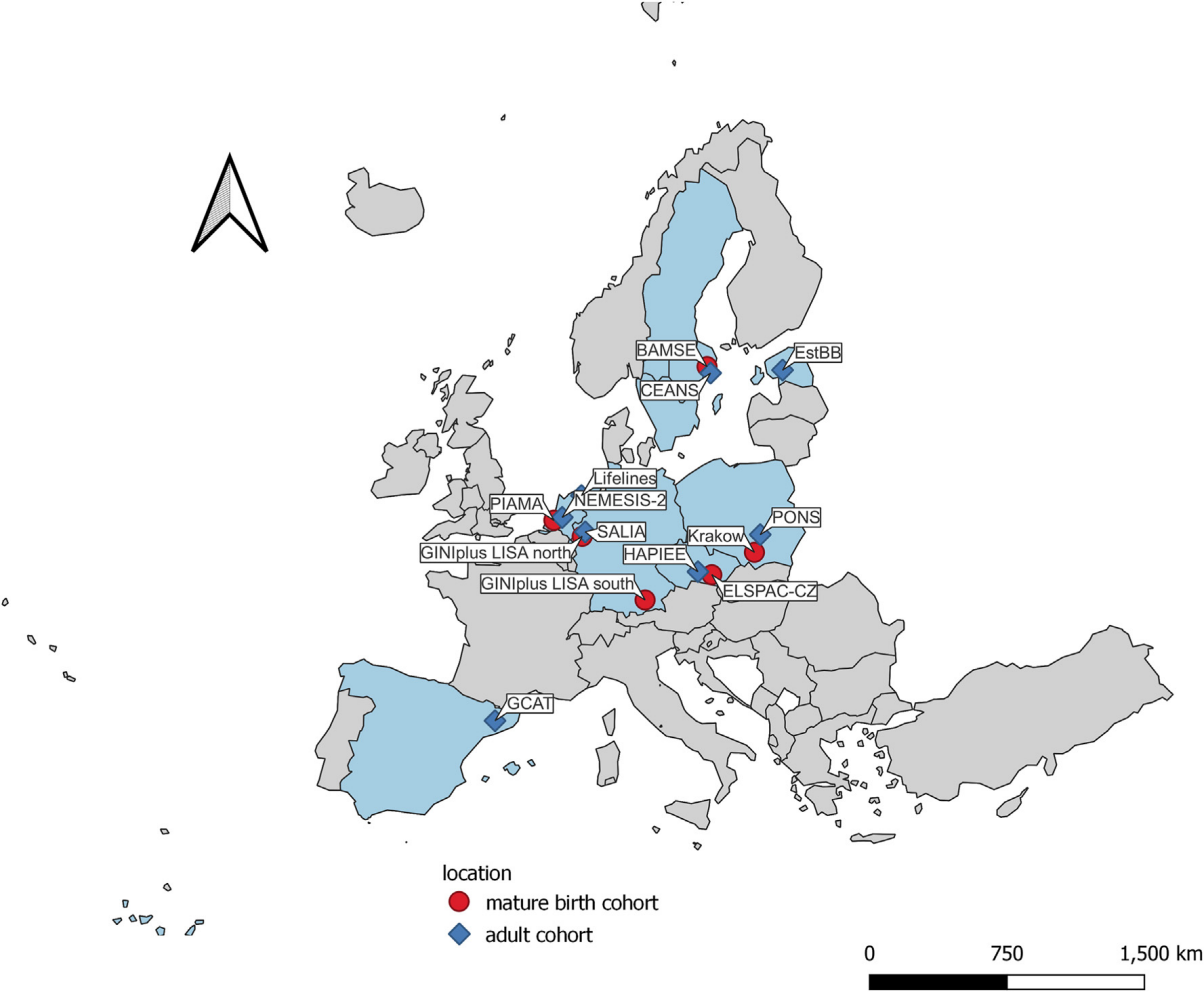
**Geographical distribution of included cohorts.** Note: The light blue areas indicate countries with participating cohorts. The red dots indicate mature birth cohorts and the blue squares indicate adult cohorts. The locations for cohorts with multiple centers may not be accurately reflected on the map due to space limitations.

We included data from 14 European cohorts, including six birth cohorts: BAMSE^15^ (Sweden), PIAMA^16^ (The Netherlands), GINIplus and LISA^17^ (Germany), Krakow birth cohort^18^ (Poland), ELSPAC-CZ^19^ (Czech Republic) and eight adult cohorts: CEANS^20^ (Sweden), GCAT^21^ (Spain), NEMESIS-2^22^ (The Netherlands), SALIA^23^ (Germany), PONS^24^ (Poland), HAPIEE^25^ (Czech Republic), Lifelines^26^ (The Netherlands), Estonian Biobank^27^ (Estonia). Geographical locations for all cohorts are shown in Fig. 1.

Additional information regarding the design and population of the included cohorts is provided in the Supplementary methods. Ethical approval was obtained from the local authorized institutional review boards, with detailed information for each cohort in the Supplementary material. In adult cohorts, we excluded participants with prevalent asthma or prevalent chronic pulmonary obstructive disease (COPD) at baseline. Data from each cohort were extracted and recoded according to a shared protocol within the EXPANSE project.^14^ The analysis for each cohort was run at the local institution using shared scripts and results were meta-analysed at Karolinska Institutet.

### Outcome assessment

Asthma outcomes were determined based on either questionnaire^28^ or linkage to local register databases (based on International Classification of Diseases, Ninth Revision (ICD-9) or Tenth Revision (ICD-10)^29^ depending on data availability). Cohort-specific definitions are described in Supplementary Table S1. In brief, six cohorts used the MeDALL definition^28^ (BAMSE, GINI, LISA, PIAMA, Lifelines, SALIA), three cohorts used the self-reported doctor-diagnosed asthma (Krakow, HAPIEE, NEMESIS-2) and five cohorts used ICD codes (ELSPAC-CZ, CEANS, EstBB, GCAT, PONS). Incidence of asthma was defined as positive when the participants without asthma at baseline fulfilled the criteria for asthma for the first time during the follow-up period.

### Exposure assessment

Environmental exposure surfaces covering the whole European region were developed within the EXPANSE project using a harmonized protocol.^30^ Three main domains of the environment, i.e., air pollution, built environment and ambient temperature were considered in the current analysis (Table 1 and Supplementary methods). Briefly, the air pollution domain covers particulate matter with median aerodynamic diameters less than 2.5 μm (PM_2.5_) and less than 10 μm (PM_10_), nitrogen dioxide (NO_2_) and ozone (O_3_); the built environment domain covers normalized differential vegetation index (NDVI), distance to green space according to Corine (GSC_DIS), distance to the blue space (BSI_DIS and BSS_DIS), imperviousness (IMP, representing the percentage of soil sealing per area unit, as a proxy of grey spaces) and artificial light at night (LAN); the ambient temperature domain covers annual and season-specific average temperature (warm season is defined as April to September, cold season is defined as October to March) averages of daily temperatures as well as the standard deviation of temperature deviations. For NDVI, IMP and LAN, 500 m buffers were used in the main analysis as a balance between capturing proximate environmental influences and broader neighbourhood effects^31^ with 300 m and 100 m buffers used in sensitivity analyses.

**Table 1:** External exposome variables in the current study.

Domain	Exposure	Spatial resolution	Temporal coverage
Air pollution	PM_2.5_	100 × 100 m	Annual (2000–2019)
	PM_10_	100 × 100 m	Annual (2000–2019)
	NO_2_	100 × 100 m	Annual (2000–2019)
	O_3_	100 × 100 m	Annual (2000–2019)
Built environment	Greenness (NDVI, 500mbuffer)	250 × 250 m	Annual average for 2000, 2005, 2010, 2015, 2020
	Greenness SD (NDVI 500mbuffer)	250 × 250 m	Annual average for 2000, 2005, 2010, 2015, 2020
	Distance to nearest green space (from Corine database)	100 × 100 m	Annual average for 2000, 2006, 2012, 2018
	Distance to blue and fresh water	100 × 100 m	Annual average for 2013
	Impervious surface (500 m buffer)	100 × 100 m	Annual average for 2006, 2009, 2012, 2015, 2018
	Light at night (500 m buffer)	100 × 100 m	Annual average for 2000, 2005, 2010, 2015, 2020
Ambient temperature	Mean temperature of the year	1 × 1 km	Annual (2003–2020)
	Standard deviation of the temperature of the year	1 × 1 km	Annual (2003–2020)
	Mean temperature of the warm season^a^	1 × 1 km	Annual (2003–2020)
	Standard deviation of the temperature of the warm season	1 × 1 km	Annual (2003–2020)
	Mean temperature of the cold season^a^	1 × 1 km	Annual (2003–2020)
	Standard deviation of the temperature of the cold season	1 × 1 km	Annual (2003–2020)

Abbreviation: nitrogen dioxide (NO_2_); particulate matter with median aerodynamic diameters <2.5 μm (PM_2.5_); particulate matter with median aerodynamic diameters <10 μm (PM_10_); Ozone (O_3_); normalized difference vegetation index (NDVI).

a Warm season is defined as April to September, cold season is defined as October to March.

For each participant, exposure at the baseline home address was extracted by cohort analysts. For exposure in the air pollution and ambient temperature domains, where annual average data were available, we assigned the annual average of the year prior to the baseline address as the main exposure. In cases where baseline investigations occurred before the availability of exposure surfaces (BAMSE, CEANS, PIAMA, GINIplusLISA, ELSPAC_CZ, SALIA), the value of the exposure at the earliest available year was assigned (2000 for air pollution and 2003 for ambient temperature). For exposure in the built environment domain, the year of available exposure value closest to the baseline year was assigned. Detailed cohort-specific baseline year and index year used for exposure assignment was shown in Supplementary Table S2.

### Covariates

Covariates were selected *a priori* based on literature^29^^,^^32^ following the disjunctive cause criterion—including variables that potentially are a cause of the exposure, the outcome, or both. Additionally, covariate definitions were chosen based on the best available information in each cohort and were harmonized across cohorts to ensure consistency. In the mature birth cohorts, the models were adjusted depending on the availability in the cohorts for time-invariant covariates (sex, maternal and paternal asthma and/or hay fever, nationality, parental education, breastfeeding, presence of older siblings at birth, daycare attendance, and maternal smoking during pregnancy) and time-varying covariates (age (indicators for time interval as dummy variable), parental smoking at home, active smoking, furry pets at home, mold/dampness at home and usage of gas cooking). In the adult cohorts, the models were adjusted depending on the availability in the cohorts for time-invariant covariates at baseline: age, sex, smoking status, body mass index (BMI), marital status, educational level, employment status, and area-level socioeconomic status.

### Statistical analysis

We applied both single exposure models and multi-exposure analyses to estimate the association between environmental exposures and asthma incidence. We included all participants with available exposure data at the baseline (birth for the birth cohorts), missing values in the covariates were coded as a separate category in the model.

For the single exposure analysis, we assessed associations using discrete time-hazard models or Cox proportional hazards models depending on the cohort data structure. For cohorts without exact asthma onset timing, we used discrete time-hazard models (also often referred to as a pooled logistic regression model), dividing follow-up into discrete intervals between questionnaires. The response variable was a binary indicator of asthma onset during each interval modelled with a logit function. For cohorts with exact asthma onset timing, we used Cox proportional hazards models with age as the time scale. The proportional hazards assumption was tested using Schoenfeld residuals. The survival time was defined from cohort entry (time origin) to the first asthma diagnosis, censoring at the end of follow-up or loss to follow-up. In the second step, we conducted random-effects meta-analyses^33^ for each exposure to combine the mean estimates (Odds ratio from discrete time hazard model and Hazard ratio from the Cox model) by cohort types.

For the cohort-specific multi-exposure analyses, we applied the k-means clustering (Hartigan and Wong algorithm^34^) for the predefined three domains (air pollution, built environment, ambient temperature) separately.^35^ To facilitate comparisons between different cohorts, the number of clusters per domain was defined as three *a priori*, and clusters were further labelled based on the most discriminatory exposures as well as consistency across cohorts. In multi-exposure association analyses, the identified clusters were assigned to each participant and further used as exposures in the regression model using the same covariates as in the single exposure analysis as well as clusters from the other two domains to obtain independent effect estimates for each domain.

To further explore the cohort-specific joint impact of the three domains of the urban environment on the risk of asthma, we calculated an “environmental risk score” for each participant using the formula^36^^,^^37^:

$${ERS}_{i}=\left(\frac{\sum_{j=1}^{n}C_{ij}\;\times \;\beta_{j}}{\sum_{j=1}^{n}\beta_{j}}\right)\;\times \;100$$

$C_{j}^{i}$ represents the cluster assignment for participants in the exposome domain j, while $\beta_{j}$ is the coefficients for this cluster obtained from the multi-exposure regression model. The score is standardized by dividing the sum of all coefficients and then multiplied by 100. A higher value of the individual weighted environmental risk score can be interpreted as exposure to a more hazardous environment. The environmental risk score was modelled as a continuous exposure with the same adjustment as the single exposure model. To explore the potential non-linear relationship, we applied natural splines in the adjusted model with three to six degrees of freedom. The results were essentially the same across the models comparing the Akaike Information Criterion values so we presented the simplest model (three degrees of freedom). For the position of the knots, we followed a quantile-based approach, placing the two internal knots at approximately the 33rd and 67th percentiles of the exposure distribution.

We explored potential modifications of associations between the environmental risk score and asthma by sex and age groups, with differences between subgroups assessed using the formula: $\beta_{1}-\beta_{2}\;\pm \;1.96\times \sqrt{{SE}_{1}^{2}\;+\;{SE}_{2}^{2}}$, where $\beta_{1}$ and $\beta_{2}$ are the coefficients of subgroups, SE1 and SE2 are the standard errors of subgroups. Age groups were defined as preschool and school age (birth to 10 years), adolescence and young adulthood (10–30 years), adulthood (30–50 years), and elderly (older than 50 years). We also estimated the population attributable fraction of asthma as the percentage of cases that could be avoided within the population if the environmental risk score would be reduced to median of the cohort—specific level. The Miettinen’s formula $PAF=\frac{P_{c}\times \left({RR}_{m-50}-1\right)}{{RR}_{m-50}}$ was used, where ${RR}_{m-50}$ is the adjusted relative risk (RR) of asthma for a binary environmental risk score (using the median as cut-off), and Pc is the prevalence of cases in the cohorts with environmental risk score larger than 50%. In the current study we used OR from the discrete time hazard model or HR from the Cox model as a proxy of the RR. 95%CIs for the PAF were calculated using the Delta method.

We conducted the following sensitivity analyses: We (1) tested the robustness of the cluster assignment by repeating the clustering using simulated datasets for each cohort (details in Supplementary methods); (2) calculated the unweighted environmental risk score by summing up the number of “harmful” clusters (defined as the clusters with the largest coefficients); (3) stratified the analysis based on change of residential addresses during the follow-up period; (4) assessed the associations with single environmental exposures at the current address of the various follow-ups instead of the baseline address in the birth cohorts; (5) tested the association using different buffer sizes (300 m and 1000 m) for NDVI, IMP and LAN; (6) conducted back-extrapolation for the air pollution exposure during 1990–2000 in selected cohorts (BAMSE, CEANS); (7) re-run the analysis using follow-up time as time axis and additionally adjusted for baseline age in CEANS, NEMESIS-2, PONS and Lifelines; (8) re-run the analysis based on the participants without any missing values in the covariates (9) additionally adjusted for indoor environmental exposures in SALIA and Lifelines (10) stratified by age 4 in the mature birth cohorts (11) using the doctor diagnosed asthma instead of the MeDALL definition in the mature birth cohorts (12) conducted leave-one-out meta-analysis to test if any single cohort was driving the association.

### Role of the funding source

The funder of the study had no role in study design, data collection, data analysis, data interpretation, or writing of the report. The corresponding author had full access to all the data in the study and had final responsibility for the decision to submit for publication.

## Results

**Fig. 2:**
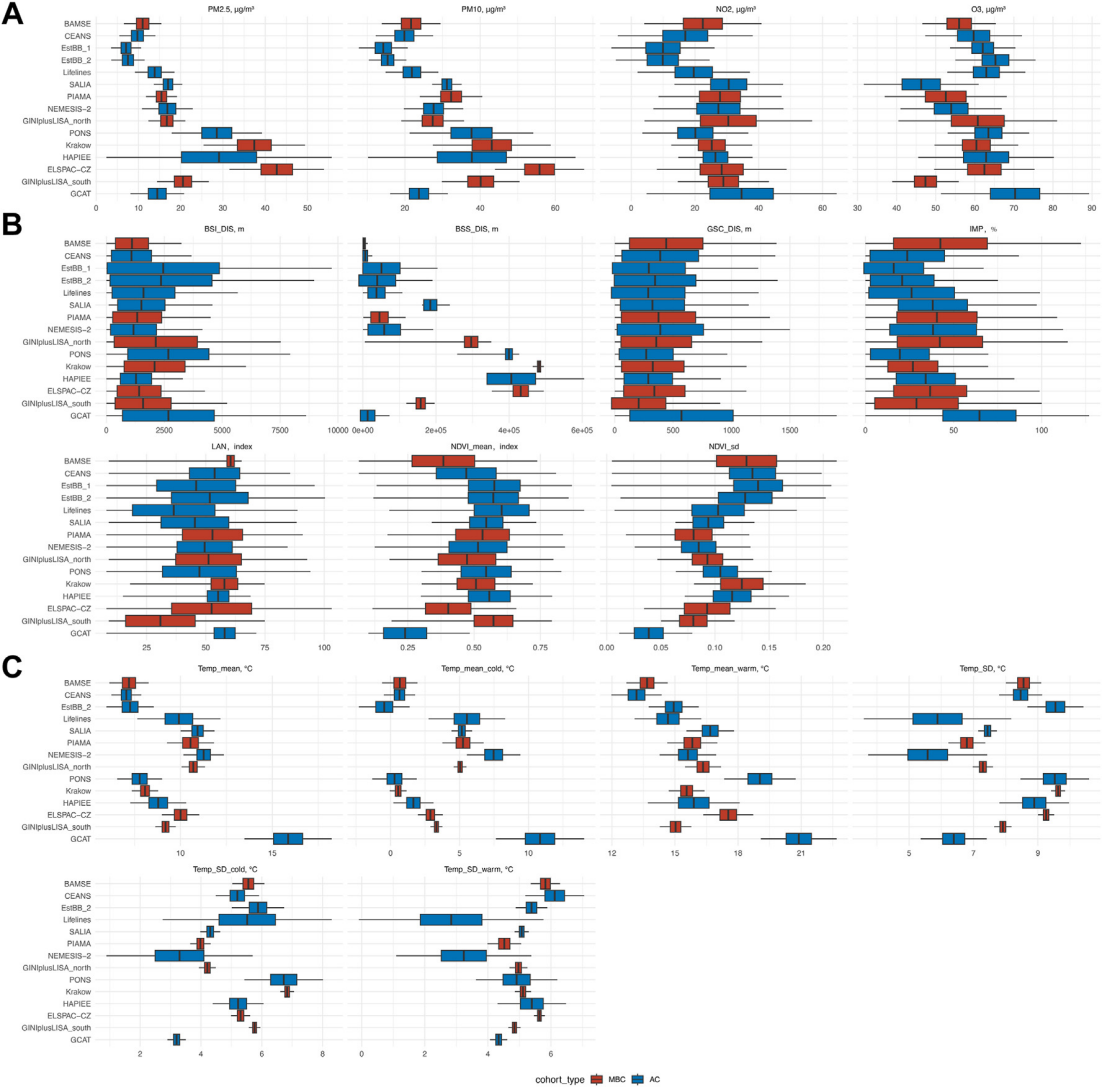
**Distribution of environmental exposures at the baseline across the included cohorts.** (A) Air pollution domain (B) Built environment domain (C) Ambient temperature domain. The cohorts were ordered based on the geographic location from north to south, with mature birth cohorts in red and adult cohorts in blue. The lower and upper bounds of the box represent the first quartile (Q1) and third quartiles (Q3). The whiskers extend from the Q1 and Q3 to the smallest and largest data points that lie within 1.5 times the interquartile range (IQR) from Q1 and Q3, respectively. Abbreviations: PM2.5, particulate matter with median aerodynamic diameters <2.5 μm; PM10, particulate matter with median aerodynamic diameters <10 μm; NO2, nitrogen dioxide; O3, Ozone; BSI_DIS, distance to the nearest inland fresh water; BSS_DIS, distance to the nearest sea; GSC_DIS, distance to the nearest green space according to the Corine database; IMP, imperviousness; LAN, light at night; NDVI_mean, average of the normalized difference vegetation index; NDVI_sd, standard deviation of the normalized difference vegetation index; Temp_mean, annual average daily temperature; Temp_mean_cold/warm, average of the daily temperature of the cold/warm season; Temp_SD, standard deviation of the daily temperature of the year; Temp_SD_cold/warm, standard deviation of the daily temperature of the cold/warm season. Note: Ambient temperature exposure is not available for EstBB_1.

Characteristics of the included participants are summarized in Table 2. In the birth cohorts, 2506 incident asthma cases were identified among 17,901 participants from birth up to young adulthood, while in the adult cohorts, 4922 incident asthma cases were identified among 331,136 participants over 2.3 million person-years. The exposure distributions for each cohort are presented in Fig. 2. Exposure to particulate matter air pollution (PM_2.5_, PM_10_) was lowest for cohorts from Northern Europe (BAMSE, CEANS, EstBB) and highest for cohorts from Poland and the Czech Republic (ELSPAC_CZ, Krakow, PONS, HAPIEE). For the built environment domain, the cohort from Spain (GCAT) had the highest IMP and lowest NDVI levels. Cohorts established in less populated areas (Lifelines, GINIplus/LISA North) had lower exposure levels of IMP and LAN. For the ambient temperature domain, the mean temperature (annual or season-specific) increased from North to South while no specific patterns were observed for temperature variations. The correlations between air pollution domains and built environment domains were similar across cohorts, with positive correlations between air pollutants (except O_3_), IMP and LAN; and negative correlations between air pollutants (except O_3_) and NDVI. Correlations between the air pollution and built environment indicators with the ambient temperature differing between the cohorts (Supplementary Fig. S1).

**Table 2:** Basic characteristics of the included cohorts.

Demographic characteristics of the mature birth cohorts						
Basic characteristics	BAMSE	ELSPAC_CZ	GINIplus/LISA North	GINIplus/LISA South	Krakow	PIAMA
N	3792	5151	1969	2889	413	3687
Incident Asthma cases, n	827	525	96	203	54	801
Follow-up periods	from birth up to 24-years old	from birth up to 19 years old	from birth up to 15 years old	from birth up to 15 years old	from birth up to 19 years old	From birth up to 20 years old
Female, n (%)	1863 (49.1%)	2493 (48.4%)	982 (49.9%)	1400 (48.5%)	206 (49.9%)	1780 (48.3%)
High parental education, n (%)	2011 (53.0%)	1740 (33.8%)	920 (46.7%)	2265 (78.4%)	266 (64.4%)	1458 (40.1%)
Parental asthma/hay fever, n (%)	1130 (29.8%)	196 (3,8%)	876 (44.5%)	1750 (60.6%)	108 (26.2%)	911 (24.7%)
Breastfeeding, n (%)	2926 (77.2%)	3321 (66.5%)	1015 (51.5%)	2385 (82.6%)	344 (83.3%)	1627 (47.0%)
Daycare attendance, n (%)	3035 (80.0%)	1268 (24.6%)	51 (2.6%)	238 (8.2%)	NA	2040 (57.7%)
Older siblings, n (%)	1836 (48.4%)	1729 (33.6%)	1061 (53.9%)	1213 (42.0%)	151 (36.6%)	1860 (50.6%)
Maternal tobacco smoking during pregnancy, n (%)	483 (12.7%)	314 (6.1%)	281 (14.3%)	353 (12.2%)	0 (0%)	626 (17.1%)
Environmental tobacco smoking, n (%)						
Early life^a^	669 (17.6%)	332 (6.4%)	735 (37.3%)	302 (10.5)	38 (9.2%)	912 (24.7%)
Most recent follow-up^b^	75/2571 (2.9%)	17/984 (1.7%)	289/1752 (16.5%)	86/2087 (4.1%)	51/189 (27.0%)	186/2127 (8.7%)
Mold and dampness, n (%)						
Early life^a^	847 (22.3%)	602 (11.7%)	283 (14.4%)	423 (14.6%)	54 (13.1%)	300 (8.2%)
Most recent follow-up^b^	255/2571 (9.9%)	314/984 (31.9%)	197/1752 (11.2%)	100/2087 (4.8%)	21/189 (11.1%)	242/2127 (11.4%)
Furry pets in the home, n (%)						
Early life^a^	488 (12.9%)	1146 (22.2%)	550 (27.9%)	670 (23.2%)	92 (22.3%)	1720 (46.8%)
Most recent follow-up^b^	538/2571 (20.9%)	507 (51.5%)	788/1752 (45.0%)	845/2087 (40.5%)	112/189 (59.3%)	877/2127 (41.2%)
Gas cooking, n (%)						
Early life^a^	477 (12.6%)	2819 (54.7%)	68 (3.5%)	217 (7.5%)	92 (22.3)	3028 (82.4%)
Most recent follow-up^b^	0/2571 (0)	NA	81/1752 (4.6%)	171/2087 (8.2%)	23/189 (12.2%)	1564/2127 (11.4%)

Demographic characteristics of the adult cohorts									
Basic characteristics	CEANS	EstBB_2	EstBB_1	GCAT	HAPIEE	Lifelines	NEMESIS-2	PONS	SALIA
N	19,357	122,408	37,890	15,604	1111	116,578	4796	12,329	1063
Incident Asthma cases, n	317	1156	1035	182	76	1865	119	127	45
Follow up years, mean (sd)	12.29 (4.01)	3.61 (0.67)	13.80 (2.82)	7.30 (1.05)	19 (2.01)	6.75 (4.20)	7.88 (2.15)	10.74 (3.25)	23.1 (3.31)
Female, n (%)	11,313 (58.4%)	80,292 (65.6%)	25,082 (66.2%)	9034 (57.9%)	661 (59.5%)	68,398 (58.7%)	2184 (45.5%)	8151 (66.1%)	1063 (100%)
Age, years, mean (sd)	56.75 (11.87)	43.34 (15.09)	41.82 (16.58)	50.88 (7.13)	56.04 (6.56)	45.2 (12.7)	44.38 (12.36)	55.26 (5.37)	73.32 (2.99)
Smoking status, n (%)									
Current smoker	4219 (21.8%)	23,049 (18.8%)	10,516 (27.8%)	2884 (18.5%)	241 (21.7%)	18,524 (15.9%)	1243 (25.9%)	2507 (20.3%)	27 (2.5%)
Former smoker	6967 (36.0%)	34,350 (28.1%)	4937 (13.0%)	6394 (41.0%)	310 (27.9%)	57,512 (49.3%)	1425 (29.7%)	4050 (32.8%)	184 (17.3%)
Never smoker	7834 (40.5%)	65,009 (53.1%)	22,437 (59.2%)	6326 (40.5%)	560 (50.4%)	40,542 (34.8%)	2128 (44.4%)	5765 (46.8%)	847 (79.7%)
BMI, kg/m^2^, mean (sd)	25.51 (4.0)	26.13 (5.04)	25.92 (5.12)	27.26 (4.53)	27.61 (4.13)	25.9 (4.18)	25.13 (4.14)	28.12 (4.62)	27.22 (4.53)
Marital status, n (%)									
Single	2624 (13.6%)	NA	NA	1854 (11.9%)	22 (2.0%)	12,147 (10.4%)	522 (10.9%)	682 (5.5%)	27 (2.5%)
Married/Living with partner	13,551 (70.0%)	NA	NA	11,278 (72.3%)	856 (77.0%)	80,446 (77.6%)	2770 (57.8%)	9793 (79.4%)	620 (58.3%)
Divorced/Separated	1618 (8.4%)	NA	NA	2029 (13.0%)	156 (14.0%)	6517 (5.6%)	1372 (28.6%)	856 (6.9%)	17 (1.6%)
Widowed	1323 (6.8%)	NA	NA	443 (2.8%)	74 (6.7%)	1426 (1.2%)	132 (2.8%)	987 (8.0%)	390 (36.7%)
Currently employed, n (%)	13,057 (67.5%)	63,670 (52.0%)	23,559 (62.2%)	11,600 (74.3%)	366 (32.9%)	90,231 (77.4%)	3686 (76.9%)	5467 (44.3%)	9 (0.8%)
High education level, n (%)	6009 (31.0%)	62,307 (50.9%)	9735 (25.7%)	6165 (39.5%)	204 (18.4%)	36,394 (31.2%)	1825 (38.1%)	3665 (29.7%)	365 (34.3%)
High area-level income, n (%)	6068 (31.3%)	86,302 (70.5%)	NA	5351 (34.3%)	NA	20,021 (17.2%)	1506 (31.4%)	8378 (68.0%)	538 (50.6%)

Data are presented as n, n(%), n/N(%) for categorical variables and mean ± sd for continuous variables.

a At baseline or during the first year of life if no baseline information is available.

b Age 24 years for BAMSE, age 19 years for ELSPAC_CZ and Krakow, age 15 years for GINIplus/LISA North and South, age 20 years for PIAMA.

In the single exposure analysis, we observed significant associations between single exposures and asthma incidence for PM_2.5_ (OR_meta_ = 1.14, 95%CI: 1.07–1.23 per 5 μg/m^3^ increase), GSC_DIS (OR_meta_ = 0.92, 95%CI:0.85–0.99 per 1 km increase) and Temp_SDC (OR_meta_ = 0.96, 95%CI:0.93–0.99 per 1 °C increase) in the adult cohorts (Supplementary Figs. S2–S4).

**Fig. 3:**
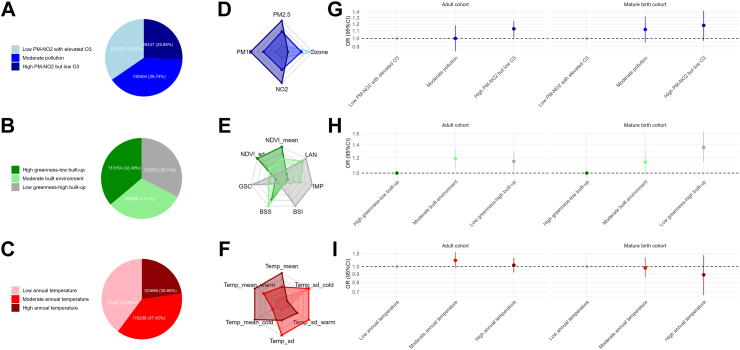
**The clusters identified within three external exposome domains and their associations with incident asthma.** Panel A, B, C are the numbers (percentages) for each cluster in air pollution, built environment, ambient temperature domains, respectively. Panel D, E, F are radar plots showing the median level of single exposure by clusters. Levels were averaged for all cohorts weighted by the sample size. Panel G, H, I show the associations between the clusters and asthma incident. Estimates were pooled using random-effect models separated by mature birth cohort and adult cohort. Estimates in the birth cohorts were adjusted depending on the availability in the cohorts for age (dummy variable), sex, parental education, parental asthma/hay fever, breastfeeding, native nationality, day care attendance, older siblings, maternal smoking, environmental tobacco smoking, mould/dampness at home, pets, use of gas cooking, active smoking and mutually adjusted for the other two environmental domains. Estimates in the adult cohorts were adjusted depending on the availability in the cohorts for age, sex, smoking status, BMI, marital status, employment status, education level, area-level SES and mutually adjusted for the other two environmental domains.

In the multi-exposure analysis using k-means clustering, the number of participants in each identified cluster is presented in Fig. 3A–C. In the air pollution domain, we identified high particulate-high NO_2_ clusters (with high levels of PM_2.5_, PM_10_, NO_2_ exposure but low levels of O_3_), moderate (intermediate levels of all pollutants), and low PM-NO2 with elevated O_3_ clusters across the cohorts (with low levels of PM_2.5_, PM_10_, NO_2_ exposure but high levels of O_3_) (Fig. 3 panel D, Supplementary Fig. S5). In the built environment domain, we identified high greenness-low built-up clusters (with high levels of NDVI, low levels of GSC, LAN and IMP), moderate (intermediate levels of all built environment exposure), and low greenness-highly built-up clusters (with low levels of NDVI, high levels of GSC, LAN and IMP), but exposure levels for blue space (BSI_DIS and BSS_DIS) were heterogeneous across the cohorts (Fig. 3 panel E, Supplementary Fig. S6). In the ambient temperature domain, clusters were labelled based on the average temperature of the year (high, moderate, low), although heterogeneity was observed for the distribution of season-specific temperature as well as temperature variations across the cohorts (Fig. 3 panel F, Supplementary Fig. S7).

When the identified clusters were used as exposure variables, we found that individuals in the high particulate matter (PM)-high NO_2_ clusters tended to have a higher risk of asthma compared to those in the low PM-NO_2_ with elevated O_3_ cluster (OR_meta_ = 1.18, 95%CI: 0.97–1.42 in birth cohorts and OR_meta_ = 1.13, 95%CI: 1.01–1.25 in adult cohorts). Low greenness-high built-up clusters as well as moderate built environment were associated with higher risks of asthma compared to high greenness-low built-up clusters (low greenness-high built-up cluster: OR_meta_ = 1.36, 95%CI: 1.14–1.64 for birth cohorts and OR_meta_ = 1.15, 95%CI:1.03–1.28 for adult cohorts). For the temperature domain, no consistent associations were observed across the cohorts (Fig. 3 Panel G–I and Supplementary Table S3 for cohort-specific results).

**Fig. 4:**
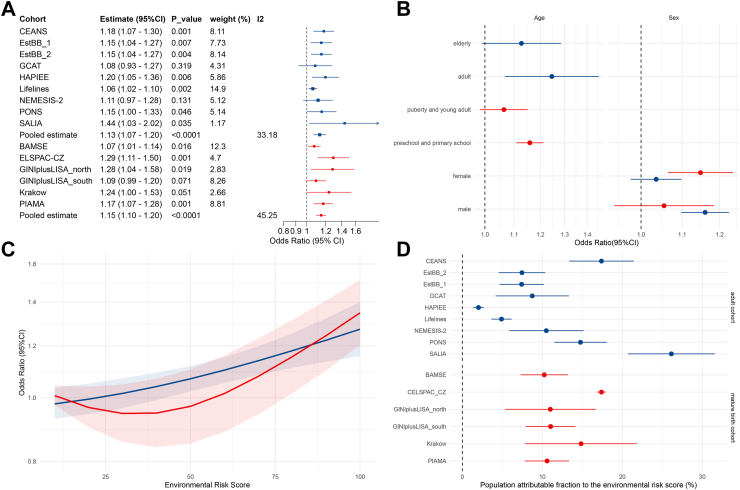
**Associations between the environmental risk score (combining three external exposome domains) and asthma incidence.** Dots or bars in blue represent results from the adult cohorts, dots or bars in red indicate results from the mature birth cohorts. (A) Cohort-specific and Meta-analysis associations between environment risk score and asthma incidence across the cohorts. Odds Ratio (OR) (95% confidence intervals (CI)) representing each standard deviation increase in the environmental risk score (B) Meta-analysis associations between environmental risk score and asthma incidence stratified by age groups and sex. (C) Meta-analysis associations between the environmental risk score and asthma incidence using natural splines with three degrees of freedom, with shaded area indicating 95% confidence interval band (D) Cohort-specific population attributable fractions for a reduction of the environmental risk score under 50%, black bars indicating the 95% confidence intervals for the population attributable fractions. Estimates in the birth cohorts were adjusted depending on the availability in the cohorts for age (dummy variable), sex, parental education, parental asthma/hay fever, breastfeeding, native nationality, day care attendance, older siblings, maternal smoking, environmental tobacco smoking, mould/dampness at home, pets, use of gas cooking, active smoking and in the adult cohorts were adjusted depending on the availability in the cohorts for age, sex, smoking status, BMI, marital status, employment status, education level, area-level SES.

In the joint exposure analysis of the three domains, the distributions of the environmental risk score were similar across cohorts (range from 0 to 100% with median around 50%, Supplementary Fig. S8) and it was consistently associated with a higher risk of asthma across cohorts with OR_meta_ = 1.13 (95%CI:1.07–1.20) for birth cohorts and 1.15 (95%CI:1.10–1.20) for adult cohorts per 20% increase in the environmental risk score (Fig. 4A).

Further, stratified analysis based on age and sex showed that females tended to be more vulnerable (OR_meta_ = 1.15 (95%CI:1.061–1.24) for females and 1.06 (0.94–1.19) for males in the birth cohorts with P value for interaction = 0.063, OR_meta_ = 1.16 (1.10–1.23) for females and 1.04 (0.98–1.10) for males in the adult cohorts with P value for interaction = 0.006, while there was no significant difference in associations between age-groups (Fig. 4B). Modelling associations of asthma with the environmental risk score using splines, we observed a general linear trend in the adult cohorts while in the birth cohorts, the exposure-response curve plateaued below 50% but increased linearly from 50% onwards (Fig. 4C). We further found that on average, 11.6% of the incident asthma cases could be attributable to environmental risk score above the median level in all cohorts (Fig. 4D).

In sensitivity analysis, (1) testing the robustness of the clustering assignments using simulated datasets, we found that the cluster assignments appeared to be highly stable for the air pollution and built environment domains and moderately stable for the ambient temperature domain (Supplementary Fig. S9A) and correlations were moderate to high (ranging from 0.61 to 0.91) for the environmental risk scores generated from simulated single cohort and pooled cohorts (Supplementary Fig. S9B). (2) Significant positive associations with risk of asthma were also observed when using the unweighted environmental score as exposure (Supplementary Table S4). (3) Stratified analyses according to moving status showed statistically significant positive associations among both movers and non-movers, with stronger estimates observed among non-movers (Supplementary Fig. S10). Similar results were observed when we (4) modelled exposures based on exposure at current addresses instead of the birth address among the birth cohorts (Supplementary Fig. S11), (5) used different buffer sizes for built environmental exposures (Supplementary Fig. S12) and (6) performed back-extrapolation for air pollution exposures during 1990–2000 (Supplementary Fig. S13) (7) used follow-up time as time axis and adjusted for baseline age had similar estimates with using age as time axis (Supplementary Fig. S14) (8) analysis based on the complete-set (Supplementary Table S5). (9) additionally adjusted for indoor environmental exposure in two adult cohorts (Supplementary Table S6) (10) stratified by age 4 in mature birth cohorts (Supplementary Table S7) (11) used alternative asthma definition in mature birth cohorts (Supplementary Table S8) (12) conducted leave-one-out meta-analysis (Supplementary Table S9).

## Discussion

In this European-wide multi-cohort exposome study, we used a clustering approach to investigate associations between multiple environmental exposures and incident asthma. In the air pollution domain, we found that exposure to clusters characterized by high levels of PM and NO_2_ were associated with higher risks of asthma among adults. In the built environment domain, we found that exposure to low levels of green space and high levels of imperviousness and light at night also were associated with higher risks of asthma. Of particular importance, we found that the joint exposure to multiple hazardous urban environmental factors, including air pollution, lack of greenness, and higher built-up areas, was consistently associated with a higher risk of incident asthma across the cohorts.

For the single exposure analysis, we did not observe strong significant associations between single environmental exposures and the risk of asthma (except for PM_2.5_ in the adult cohorts). Air pollution is the most studied among the included environmental exposures. For the participating cohorts that have reported associations between air pollution exposure and incident asthma previously (for example PIAMA, BAMSE, GINIplus/LISA and CEANS), associations in the current study are consistent with previous findings.^29^^,^^32^ By including additional cohorts in the present study, the association between air pollution and asthma was not evident in the single-exposure analysis model but became more pronounced in the clustering-based multi-exposure models. This could be potentially due to the confounding or synergistic effects between the different single exposures and therefore, an exposome-based approach should preferably be adopted by future studies. For the built environment domain, our findings are in line with the reported association of green and grey spaces with asthma in the Spanish INMA study.^38^ It has been reported that urban grey spaces tend to have higher levels of airborne allergens, which could increase the risk of developing asthma.^39^ Exposure to artificial light at night may disrupts the body’s natural circadian rhythms and lead to melatonin dysregulation, which may be relevant with asthma.^40^ For long-term (annual and seasonal) average temperature and temperature variability, a recent systematic review included six studies on long-term temperature indicators and respiratory outcomes (mortality, hospital admission, chronic bronchitis) and found the meta-analysed results to be largely null.^41^ It was speculated that the effect of small variations in long-term temperature exposure is difficult to capture by a small cohort within cities or countries.

Evidence regarding the association between multiple environmental exposures and asthma remains limited. In the French NutriNet-Santé Study, adults living in highly built-up urban areas with low greenspace were found to have worse asthma control.^12^ In the Dutch Generation R study, clusters characterized by high levels of air pollution, noise, walkability and low levels of nature space during pregnancy were associated with higher odds of preschool wheezing of the child.^11^ In the HELIX study (combined data from France, Greece, Lithuania, Norway, Spain and the United Kingdom), the pregnancy exposure profiles including high levels of exposure to relative humidity, air pressure and temperature were associated with higher odds of ever-asthma and wheezing.^13^ The current study largely extends the previous evidence in terms of: 1) incorporating asthma incidence with a longitudinal design which helps to clarify the role of environmental factors in asthma inception; 2) by including both mature birth cohorts and adult cohorts, our study was able to cover the entire life-course and to show that the multiple environmental exposures affect asthma in all age groups ubiquitously; 3) the clusters derived in the previous studies were study/location-specific and therefore, the results cannot be readily compared between studies and limited in generalization of results. By comparing the cluster memberships among the simulated single cohorts and pooled cohort datasets, we showed moderate to good similarities for the clusters, especially for the air pollution and built environment domain across the cohorts, indicating that these exposure patterns (for example higher built-up imperviousness, light at night and low green space) were consistent across cohorts.

We observed significantly stronger associations of the environment risk score with asthma onset among females compared to males. This finding is in line with previous results on sex differences in environmental exposure effects,^42^ although findings are not consistent for respiratory outcomes.^43^ Future studies are needed to investigate potential biological mechanisms behind sex differences. In age-stratified analysis, we did not find the association of the environment risk score with asthma to be restricted to specific age groups, indicating that the benefits from optimal urban planning and reducing harmful environmental exposures may benefit respiratory health across all life stages.^4^ We observed stronger association estimates of the environmental risk score among non-movers compared to movers in both mature birth cohorts and adult cohorts (Supplementary Fig. S10). This finding likely reflects the importance of cumulative environmental exposure in the development of asthma. For non-movers, the baseline exposure more accurately represents long-term exposure, whereas for movers, a single baseline measurement may not capture the variation in exposure over time. Consequently, the exposure misclassification in movers could bias the estimates towards the null. It would be valuable for future research to investigate the time-varying multi-exposure setting, ideally with a more specific design and modelling^44^ such as trajectory analysis^45^ or a mover-based natural experiment design.^35^

Our findings have important public health implications as on average 11.6% of incident asthma cases could be attributed to the environment risk score above their cohort-specific median level. A higher environmental risk score in general represents higher exposure to air pollutants (PM and NO_2_), lower exposure to greenness and higher exposure to built-up areas (IMP and LAN), although the specific contributions of these factors vary across cohorts. This variability highlights the need for tailored intervention strategies that address the unique environmental challenges of each region of the cohorts. Our results underscore the importance of incorporating multiple environmental exposures into urban planning, including the identification of local “hotspots” where harmful exposures overlap. While harmful environmental exposures are a global issue, solutions must also be identified on the local/small-area level and adapted to effectively mitigate specific exposure patterns.^6^ It should be noted that the estimation of PAF was based on assumptions including causal relationships, other risk factors unaffected and realistic interventions, which may be violated in real-life settings (for example increasing the urban green space may influence surrounding people’s physical activity behavior) and lead to biased estimates.

Strengths of the current study include the large sample size, the length of the follow-up time, the longitudinal assessment of the outcome, the standardized exposure assessment on the individual address basis and the harmonization of confounder data between the cohorts. We also acknowledge several limitations. First, due to the observational study design, causal relationships cannot be firmly drawn from the current study. We acknowledge the existence of unmeasured confounding factors, although many potential confounders have been adjusted for using lifestyle, indoor environment and both individual- and area-level socioeconomic status variables. Residual confounding due to measurement errors in the confounders may also exist. For example, the smoking variable (current/former/never smoker) may not fully capture smoking status. We also used random-effects meta-analysis when pooling the estimates to better account for the differences between cohorts that are not adjusted for in the model. Second, asthma diagnosis in young children remains very challenging, and that it is difficult to disentangle asthma disease from wheezing symptoms in this age group. But sensitivity analysis in the mature birth cohorts showed significant associations for the joint exposure among children above 4 years of age. Third, for cohorts with baseline recruitment before 2000, the earliest available exposure was assigned to the baseline residential addresses. This was done based on the assumption that the spatial contrast remained stable over the years. For air pollution exposure, we tested this assumption by comparing the associations with and without back-extrapolation in two cohorts (BAMSE and CEANS) and the results supported this assumption (Supplementary Fig. S12), but data are lacking for the exposures in the built environment and ambient temperature domains. We acknowledge the potential limitations that asthma definitions were not harmonized across all cohorts, but the association estimates of environmental risk score were consistently positive across the cohorts and using alternative definitions in four mature birth cohorts yielded similar results (Supplementary Table S8). We also lack the data to further explore the associations between urban environment exposure and asthma subtypes and endotypes. In adults, the absolute number of incident asthma cases in some of the cohorts was also relatively small. For data availability reasons, we were unable to include other relevant urban environmental factors, such as road traffic noise, walkability, which could be of interest for future studies to estimate the joint impact of the total environment. While this study used NDVI as an indicator of greenness exposure and examined joint exposure associations, the potential modification/mediation roles of NDVI (e.g., on air pollution) and seasonal variations in greenness warrant further investigation in future studies. Finally, the estimates derived in the current study (odds ratios and hazard ratios) are not conceptually equivalent to risk ratios, even though in our study the incident asthma outcome is relatively rare. In conclusion, joint exposure to multiple harmful urban environmental exposures including air pollution, lack of green space, high imperviousness and light at night is associated with higher risks of asthma incidence among both children and adults.

## Contributors

ZY contributed to Conceptualisation, Data curation, Formal analysis, Investigation, Visualisation, Writing original draft and Write reviewing & editing. SK contributed to Data curation, Formal analysis, Investigation and Write reviewing & editing. MO, NB, ML, RM, DS contributed to Formal analysis and Write reviewing & editing. PG, MM, PM, SD, OM, JK, KW, YS, AB, AD contributed to Data curation and Write reviewing & editing. HK contributed to Data curation, Formal analysis and Write reviewing & editing. JK contributed to Data curation, Project administration, Supervision and Write reviewing & editing. KF, RdeC contributed to Data curation, Supervision and Write reviewing & editing. PP contributed to Resources. MH, PL, JV, RV, GP, AS, PD, CT contributed to Conceptualisation and Write reviewing & editing. AP, HP contributed to Data curation, Funding acquisition and Write reviewing & editing. MS contributed to Data curation, Funding acquisition, Investigation, Resources and Write reviewing & editing. TS, AB, ASM contributed to Funding acquisition, Resources and Write reviewing & editing. JL contributed to Resources and Write reviewing & editing. KH contributed to Data curation, Resources, Supervision and Write reviewing & editing. UG contributed to Conceptualisation, Data curation, Funding acquisition, Resources, Supervision and Write reviewing & editing. GK contributed to Conceptualisation, Data curation, Funding acquisition, Resources and Write reviewing & editing. JV contributed to Conceptualisation, Resources and Write reviewing & editing. CHE contributed to Conceptualisation and Methodology. OG contributed to Conceptualisation, Data curation, Supervision and Write reviewing & editing. AP contributed to Conceptualisation, Funding acquisition, Resources and Write reviewing & editing. SKM contributed to Conceptualisation, Methodology and Write reviewing & editing. EM contributed to Conceptualisation, Data curation and Write reviewing & editing. ZY, SKM, EM had access to raw data, SKM verified the data and EM had final responsibility for the decision to submit for publication.

## Data sharing statement

The datasets analysed in the current study are not publicly available, but the derived data supporting the findings of this study are available from the corresponding author (EM) on reasonable request.

## Editor note

The Lancet Group takes a neutral position with respect to territorial claims in published maps and institutional affiliations.

## Declaration of interests

GHK reports research grants from ZON-MW (VICI grant), H2020 (Prominent), Netherlands Lung Foundation, Vertex, Ubbo Emmius Foundation, TEVA the Netherlands, outside the submitted work (Money to institution). His institution received financial compensation for advisory board meetings to Astra Zeneca, and lectures from Boehringer-Ingelheim, Sanofi and Astra Zeneca. EM reports advisory board fees from ALK and AstraZeneca; and lecture fees from ALK, AstraZeneca, Chiesi and Sanofi outside the submitted study. PL reports travel expenses for invited lecture at conference on air pollution and health effects from Fondazione Menarini.

## Appendix ASupplementary data

Supplementary Figures S1–S14 and Tables S1–S9

EstBB bannerauthor for pubmed
