# Supplementary material for: External exposome and incident asthma across the life course in 14 European cohorts: a prospective analysis within the EXPANSE project
Source: Lancet Reg Health Eur. 2025 May 15;54:101314. doi: 10.1016/j.lanepe.2025.101314 (PMC12266180; doi:10.1016/j.lanepe.2025.101314)
Supplement: Supplementary Figures S1–S14 and Tables S1–S9 [file mmc2.docx]

**External exposome and incident asthma across the life course in 14 European cohorts: the EXPANSE project**

Contents

[Contents 1](#_Toc193636443)

[Description of the participating mature birth cohorts 3](#_Toc193636444)

[Description of the participating adult cohorts 6](#_Toc193636445)

[Exposure assessment 10](#_Toc193636446)

[Simulation for testing the cluster assignments 11](#_Toc193636447)

[Cohort-specific funding and acknowledgements 12](#_Toc193636448)

[Supplemental Table 1 Asthma definitions in the cohorts 15](#_Toc193636449)

[Supplemental Table 2 Information for adjusted covariates and baseline year in each cohort 17](#_Toc193636450)

[Supplemental Table 3 Associations between the urban environment clusters with asthma incidence across the cohorts 19](#_Toc193636451)

[Supplemental Table 4 Associations between unweighted environmental score with asthma incidence across cohorts 20](#_Toc193636452)

[Supplemental Table 5 Percentage of missing category in the covariates across cohort and associations between environmental risk score with asthma incidence across cohorts using the complete-set dataset 21](#_Toc193636453)

[Supplemental Table 6 Associations between environmental risk score with asthma incidence in SALIA and Lifelines additionally adjusted for indoor environmental exposure 23](#_Toc193636454)

[Supplemental Table 8 Comparison of the association between environmental risk score and asthma incidence using MeDALL definition and doctor diagnosis only 25](#_Toc193636455)

[Supplemental Table 9 Leave-one-out meta-analysis for the association between environmental risk scores with asthma incidence 26](#_Toc193636456)

[Supplemental Figure 1 Correlations between urban environmental exposures at baseline addresses 27](#_Toc193636457)

[Supplemental Figure 2 Meta-analysis of single exposure models with asthma incidence in the air pollution domain 28](#_Toc193636458)

[Supplemental Figure 3 Meta-analysis of single exposure models with asthma incidence in the built environment domain 29](#_Toc193636459)

[Supplemental Figure 4 Meta-analysis of single exposure models with asthma incidence in the ambient temperature domain 30](#_Toc193636460)

[Supplemental Figure 5 Cluster-based distribution of air pollution exposures across cohorts 31](#_Toc193636461)

[Supplemental Figure 6 Cluster-based distribution of built environment exposure across cohorts 33](#_Toc193636462)

[Supplemental Figure 7 Cluster-based distribution of temperature exposure across cohorts 35](#_Toc193636463)

[Supplemental Figure 8. Distributions of the environmental risk scores across the cohorts 37](#_Toc193636464)

[Supplemental Figure 9. Comparison of cluster assignment using adjusted rand index (A) and Pearson’s correlation index for the environment risk score (B) between the cohort-specific and pooled simulated datasets. 38](#_Toc193636465)

[Supplemental Figure 10 Association between the weighted environmental score with asthma incidence stratified by residential change during the follow-up period. 39](#_Toc193636466)

[Supplemental Figure 11 Comparison of the meta-analyzed association between single exposure based on baseline addresses and current addresses in the mature birth cohorts 40](#_Toc193636467)

[Supplemental Figure 12 Sensitivity analysis for the built environmental exposure domain with different buffer sizes 41](#_Toc193636468)

[Supplemental Figure 13 Sensitivity analysis for air pollution exposure based on back-extrapolations 43](#_Toc193636469)

[Supplemental Figure 14 Sensitivity analysis for using follow-up time as time axis in survival analysis in four adult cohorts 44](#_Toc193636470)

**Supplemental Methods**

# Description of the participating mature birth cohorts

**BAMSE**

The BAMSE (Children, Allergy, Environment, Stockholm, Epidemiology (in Swedish) study is an ongoing population-based birth cohort in Stockholm, Sweden, including 4089 newborns in 1994-1996. Details on study design, recruitment procedure and data collection have been provided elsewhere. Follow-ups with questionnaires were conducted at the ages of 1, 2, 4, 8, 12, 16 and 24 years, while at ages of 8, 16, 24 clinical examinations were also conducted. The study was approved by the Swedish Ethical Review Authority and all participants or their caregivers during childhood, gave written informed consent.

Reference for study design and data collection:

- Wickman, M., Kull, I., Pershagen, G., Nordvall, S.L., 2002. The BAMSE project: presentation of a prospective longitudinal birth cohort study. Pediatr Allergy Immunol 13, 11–3. https://doi.org/10.1034/j.1399-3038.13.s.15.10.x
- Melen, E., Bergstrom, A., Kull, I., Almqvist, C., Andersson, N., Asarnoj, A., Borres, M.P., Georgellis, A., Pershagen, G., Westman, M., van Hage, M., Ballardini, N., 2020. Male sex is strongly associated with IgE-sensitization to airborne but not food allergens: results up to age 24 years from the BAMSE birth cohort. Clin Transl Allergy 10, 15. https://doi.org/10.1186/s13601-020-00319-w

**ELSPAC_CZ**

The European Longitudinal Study of Pregnancy and Childhood (ELSPAC) is a long-term research project that examines the health and development of children from birth to adulthood. It was initiated by the World Health Organization (WHO) in the early 1990s and includes several European countries, including the Czech Republic referred to as ELSPAC-CZ, as well as the ALSPAC study in the UK.

In the Czech Republic, the study began in 1991 enrolling 5,151 children born in Brno and Znojmo regions. It collects comprehensive data on prenatal and postnatal health, environmental exposures, nutrition, and psychosocial factors. The goal was to understand how various biological, environmental, and social influences shape health and well-being over time. The ELSPAC-CZ study provides valuable insights into public health, childhood development, and the long-term effects of early-life exposures.

The secondary use of all ELSPAC study data was approved by the (C)ELSPAC Ethics Committee (Ref. No. ELSPAC/EK/1/2014, date 09/17/2014).

Reference for data collection and study design:

- Piler P, Kandrnal V, Kukla L, Andrýsková L, Švancara J, Jarkovský J, Dušek L, Pikhart H, Bobák M, Klánová J. Cohort Profile: The European Longitudinal Study of Pregnancy and Childhood (ELSPAC) in the Czech Republic. Int J Epidemiol. 2017 Oct 1;46(5):1379-1379f. doi: 10.1093/ije/dyw091. PMID: 27380795; PMCID: PMC5837270.

**GINIplus/LISA North & South**

The GINIplus (German Infant Nutritional Intervention plus environmental and genetic influences on allergy development) and LISA (Influence of Lifestyle factors on the development of the Immune System and Allergies) studies are prospective population-based birth cohorts from East and West Germany. In GINIplus, 5,991 healthy full-term newborns of European ancestry were enrolled between 1995 and 1998, in LISA, 3,097 between 1997 and 1999, across four study areas in Germany (Munich, Wesel, Leipzig, and Bad Honnef). Ethical approval for both studies was obtained from local ethics committees (Bavarian General Medical Council, Medical Council for North-Rhine-Westphalia and the University of Leipzig), and all participants or their caregivers gave written informed consent. Details on the study design are provided elsewhere.

For GINIplus and LISA, which have nearly identical study designs, data were pooled and then analysed separately for the urban area Munich (GINIplus/LISA South) and the rural area Wesel (GINIplus/LISA North).

Reference for data collection and study design:

- Heinrich J, Brüske I, Cramer C, Hoffmann U, Schnappinger M, Schaaf B, von Berg A, Berdel D, Krämer U, Lehmann I, Herbarth O, Borte M, Grübl A, Bauer CP, Beckmann C, Behrendt H, Ring J, Koletzko S. GINIplus and LISAplus - Design and selected results of two German birth cohorts about natural course of atopic diseases and their determinants. Allergol Select. 2017 Aug 4;1(1):85-95. doi: 10.5414/ALX01455E. PMID: 30402607; PMCID: PMC6040001.
- Berg A, Krämer U, Link E, Bollrath C, Heinrich J, Brockow I, Koletzko S, Grübl A, Filipiak-Pittroff B, Wichmann HE, Bauer CP, Reinhardt D, Berdel D; GINIplus study group. Impact of early feeding on childhood eczema: development after nutritional intervention compared with the natural course - the GINIplus study up to the age of 6 years. Clin Exp Allergy. 2010 Apr;40(4):627-36. doi: 10.1111/j.1365-2222.2009.03444.x. Epub 2010 Jan 14. PMID: 20082618.
- Heinrich J, Bolte G, Hölscher B, Douwes J, Lehmann I, Fahlbusch B, Bischof W, Weiss M, Borte M, Wichmann HE; LISA Study Group. Allergens and endotoxin on mothers' mattresses and total immunoglobulin E in cord blood of neonates. Eur Respir J. 2002 Sep;20(3):617-23. doi: 10.1183/09031936.02.02322001. PMID: 12358337.

**Krakow birth cohort**

The Krakow Birth Cohort has been established in Krakow, Poland in years 2000-2003 as the result of collaboration between the Jagiellonian University in Krakow and Columbie University in New York. The main aim of the study was to assess the health impact of prenatal exposure to outdoor/indoor

air pollution in infants and children from the Kraków inner city area. Pregnant women living in Krakow, Poland were recruited from ambulatory prenatal clinics in their first or second trimesters of pregnancy. Only women 18–35 years of age, who claimed to be non-smokers, with singleton pregnancies, with no history of illicit drug use and HIV infection, free from chronic diseases such as diabetes or hypertension, and who had resided in Krakow for at least one year prior to pregnancy were eligible for the study.

Upon enrollment, a detailed questionnaire was administered to each woman to solicit information on demographic data, house characteristics, medical and reproductive history, occupational hazards, and passive smoking. During the 24^th^-26^th^ week of pregnancy, the individual personal measurements of ambient air pollution by PM_2.5_ and polycyclic aromatic hydrocarbons were performed covering 48-hour time period. In a subsample of the pregnant women, the air pollution measurements covered also indoor and outdoor measurements with the use of the same equipment and during the same time period as personal ones. A total of 505 enrolled pregnant women gave birth between January 2001 and February 2004. The follow-up visits were scheduled, every three months in the first two years of the newborn’s life, every 6 months later up to age of five years, and yearly up to the age of nine. The later, follow-ups were performed in years 2015-2016 and 2018- 2019. During each follow-up visit a detailed standardized face-to-face interview was conducted regarding the child's/teenager's health, with particular emphasis on respiratory diseases. In addition to the questionnaire data, the psychomotor development data were collected, and starting with age of four years spirometric measurements were performed (all spirometric measurements were carried out with a computerized PC QRS Card Spirometer with incentive display software).

The study was approved by The Bioethics Committee of the Jagiellonian University.

Reference for data collection and study design

- Jedrychowski W, Whyatt RM, Camann DE, Bawle UV, Peki K, Spengler JD, et al. Effect of prenatal PAH exposure on birth outcomes and neurocognitive development in a cohort of newborns in Poland. Study design and preliminary ambient data. Int J Occup Med Environ Health 2003;16:21–9.
- [Jedrychowski](https://pubmed.ncbi.nlm.nih.gov/?term=Jedrychowski%20WA%5BAuthor%5D) WA, Perera FP, Maugeri U, [Mroz](https://pubmed.ncbi.nlm.nih.gov/?term=Mroz%20E%5BAuthor%5D) E, Klimaszewska-Rembiasz M, Flak E, Edwards S, Spengler JD. Effect of prenatal exposure to fine particulate matter on ventilatory lung function of preschool children of nonsmoking mothers. Krakow inner city birth cohort prospective study. Paediatr Perinat Epidemiol. 2010; 24(5): 492–501, doi: [10.1111/j.1365-3016.2010.01136.x](https://doi.org/10.1111%2Fj.1365-3016.2010.01136.x)
- Majewska R, Pac A, Mróz E, Spengleer J, Camann D, Mrozek-Budzyn D, Sowa A, Jacek R, Wheelock K, Perera F. Lung function growth trajectories in non-asthmatic children aged 4–9 in relation to prenatal exposure to airborne particulate matter and polycyclic aromatic hydrocarbons – Krakow birth cohort study. Environ Res. 2018; 166: 150-157, doi: 10.1016/j.envres.2018.05.037.

**PIAMA**

PIAMA is a population-based prospective birth cohort study with detailed descriptions published previously. In brief, pregnant women were recruited from communities in different regions of the Netherlands in 1996–1997, and their children (N = 3963) were followed up by repeated questionnaire surveys (parental-completed at age 3 months and then annually until age 8, from age 11 onwards every 3 years parent- and -participant completed, and at age 20 participant completed only) including questions about health, demographic factors and risk factors for asthma and respiratory health. The institutional review boards of the participating institutes approved the study protocol and written informed consent was obtained from the parents or legal guardians of all participants.

Reference for data collection and study design:

1. Brunekreef, B., Smit, J., de Jongste,J., Neijens, H., Gerritsen, J., Postma, D., Aalberse, R., Koopman, L., Kerkhof, M., Wijga, A., van Strien, R., 2002. The prevention and incidence of asthma and mite allergy (PIAMA) birth cohort study: design and first results. Pediatr. Allergy Immunol. 13, 55–60.

2. Wijga, A.H., Kerkhof, M., Gehring, U., de Jongste, J.C., Postma, D.S., Aalberse, R.C., Wolse, A.P., Koppelman, G.H., van Rossem, L., Oldenwening, M., Brunekreef, B., Smit, H.A., 2014. Cohort profile: the prevention and incidence of asthma and mite allergy (PIAMA) birth cohort. Int. J. Epidemiol. 43, 527–535.

# Description of the participating adult cohorts

**CEANS**

The CEANS (Cardiovascular Effects of Air Pollution and Noise in Stockholm study) combined data from four sub-cohorts: The Screening Across the Lifespan Twin Study (SALT) sampled 7,043 individuals from the Swedish Twin Register born 1958 and earlier, who lived in Stockholm County [1]. The Stockholm Diabetes Preventive Program (SDPP) is a population-based prospective study of 7,949 subjects aged 35–54 years [2]. The SIXTY subcohort consists of a random population sample of one-third of all men and women living in Stockholm County turning 60 years between August 1997 and March 1999 [3]. Lastly, The Swedish National Study of Aging and Care in Kungsholmen (SNAC-K) randomly sampled individuals 60+ years of age from a central area in Stockholm [4]. All participants resided in Stockholm County, Sweden.

Reference for data collection and study design:

1. Lichtenstein, P. et al. The Swedish Twin Registry in the Third Millennium: An Update. Twin Res.

Hum. Genet. 9, 875–882 (2006).

1. Eriksson, A. K. et al. Psychological distress and risk of pre-diabetes and Type 2 diabetes in a

prospective study of Swedish middle-aged men and women. Diabet. Med. 25, 834–842 (2008).

1. Wändell, P. E., Wajngot, A., de Faire, U. & Hellénius, M. L. Increased prevalence of diabetes

among immigrants from non-European countries in 60-year-old men and women in Sweden.

Diabetes Metab. 33, 30–36 (2007).

1. Lagergren, M. et al. A longitudinal study integrating population, care and social services data.

The Swedish National study on Aging and Care (SNAC). Aging Clin. Exp. Res. 16, 158–168

(2004).

**Estonian Biobank**

The Estonian Biobank [1,2,3] is a volunteer-based biobank of Estonia, currently including more than 210,000 individuals. Details on biobank, recruitment procedure and data collection have been provided elsewhere [2]. The Estonian Biobank database is regularly linked with national registries, hospital and national health insurance fund databases. The activities of the EstBB are regulated by the Human Genes Research Act, which was adopted in 2000 specifically for the operations of the EstBB. All participants have signed an informed consent form during recruitment. Individual level data analysis in the EstBB was carried out under ethical approvals 1.1-12/3435 (08.12.2020), 1.1-12/1021 (13.04.2021), 1.1-12/1021 (14.12.2021), 1.1-1/3452 (20.10. 2022), 1.1-12/1086 (13.03.2023), 1.1-12/4367 (07.12.2023) from the Estonian Committee on Bioethics and Human Research (Estonian Ministry of Social Affairs), using data according to release application 3-10/GI/31961 from the Estonian Biobank.

Data analysis was carried out in part in the High-Performance Computing Center of University of Tartu.

The sub-cohort EstBB_1 is formed from over 41 000 participants who joined the Estonian Biobank before 2018, from 2002 to 2017. For EstBB2 sub-cohort questionnaires were conducted at the time of recruitment at the recruitment office or GP and for a subset of biobank participants, additional questionnaires have been filled as part of subsequent studies. EstBB_1 sub-cohort participants who joined the biobank several years ago may have a total of up to four different values of questionnaire answers. The answer of questionnaire is used which is closest to the beginning of the observation, 2014. For this study EstBB_1 sub-cohort is observed from 2014 to 2022, follow-ups from the electronic health records and death registry are conducted every year.

The sub-cohort EstBB_2 is formed from over 128 000 participants who joined the Estonian Biobank from 2018. For EstBB_2 sub-cohort questionnaires were sent electronically after recruitment. For this study EstBB_2 sub-cohort is observed from 2018 to 2022, via regular follow-up from electronic health records and death registry.

Author list for Estonia Biobank Research team:

| First name | Last name | Affiliation |
| --- | --- | --- |
| Andres. | Metspalu | Estonian Genome Centre, Institute of Genomics, University of Tartu, Tartu, Estonia |
| Lili. | Milani | Estonian Genome Centre, Institute of Genomics, University of Tartu, Tartu, Estonia |
| Tõnu | Esko | Estonian Genome Centre, Institute of Genomics, University of Tartu, Tartu, Estonia |
| Mait | Metspalu | Estonian Biocentre, Institute of Genomics, University of Tartu, Tartu, Estonia |

Reference for data collection and study design:

1.Estonian Biobank. The University of Tartu Institute of Genomics. 2021. <https://genomics.ut.ee/en/content/estonian-biobank>

2.Leitsalu L, Haller T, Esko T, et al. Cohort Profile: Estonian Biobank of the Estonian Genome Center, University of Tartu. *Int J Epidemiol*. 2015;44(4):1137-1147. doi:10.1093/ije/dyt268

3. https://www.medrxiv.org/content/10.1101/2024.09.22.24313964v1.article-info

**GCAT|Genomes for Life**

The GCAT Cohort is a population-based study from Catalonia, established with active follow-up to explore various health outcomes. Between 2014 and 2018, participants aged 40 to 65 were voluntarily recruited, primarily through blood and tissue donation centers of the Blood and Tissue Bank of Catalonia (BST), though participation was not limited to regular donors, only living in Catalonia was the restriction criteria to participate. All participants provided informed consent, allowing access to their electronic health records (EHRs) from the public healthcare system for yearly passive follow-up (2010-2024), under the PADRIS framework. Active follow-up has been conducted in 2018, 2020, 2021, 2023. They also agreed to be contacted regularly to provide updates on lifestyle factors and additional health information.

Data collection included anthropometric measurements, blood samples, and comprehensive self-reported questionnaires covering sociodemographic factors, lifestyle habits, geocoded exposures, and health conditions. For a subset of participants, multi-omics data has been generated, including genotyping (SNP-Array), whole-genome sequencing (WGS), metabolomics, proteomics, and epigenetic information. Participants maintain the right to opt-out or withdraw consent for specific aspects of the research.

The study protocol is outlined in Obón-Santacana et al. (2018) and flagship paper (*1-3*). The GCAT study was approved by the Germans Trias i Pujol University Hospital Ethical Committee (PI-13-020).

1. I. Galvan-Femenia et al., Multitrait genome association analysis identifies new susceptibility genes for human anthropometric variation in the GCAT cohort. J Med Genet 55, 765-778 (2018).

2. M. Obon-Santacana et al., GCAT|Genomes for life: a prospective cohort study of the genomes of Catalonia. BMJ Open 8, e018324 (2018).

3. J. Valls-Margarit et al., GCAT|Panel, a comprehensive structural variant haplotype map of the Iberian population from high-coverage whole-genome sequencing. Nucleic Acids Res 50, 2464-2479 (2022).

**HAPIEE**

Health, Alcohol and Psychosocial factors In Eastern Europe (HAPIEE) is the ongoing longitudinal cohort study that was established to investigate the effects of dietary factors, alcohol consumption and psychosocial factors on the health. The baseline investigation was carried out in 2002-2005 with a total of 29,847 recruited women and men aged 45-69 years. The participants were recruited in four Eastern European countries, including the Czech Republic, Poland, Lithuania and Russia. Data collection included anthropometric and blood pressure measurements, spirometry, grip strength, walk speed, examination of cognitive functions, and blood sampling. The baseline survey was funded by Wellcome Trust and McArthur Foundation. For this particular study, only data from the Czech arm of the HAPIEE study was utilized. The follow-up investigation was conducted in 2023-2024 and was supported by the RECETOX Research Infrastructure and by the NPO „Systemic Risk Institute“ (LX22NPO5101), funded by European Union – Next Generation EU (Ministry of Education, Youth and Sports, NPO: EXCELES). All participants provided informed consent, and the institutional ethics committees approved the study.

1. Peasey A, Bobak M, Kubinova R, Malyutina S, Pajak A, Tamosiunas A, Pikhart H, Nicholson A, Marmot M. Determinants of cardiovascular disease and other non-communicable diseases in Central and Eastern Europe: rationale and design of the HAPIEE study. BMC Public Health. 2006; 6:255. doi: 10.1186/1471-2458-6-255.

**Lifelines**

Lifelines is a multi-disciplinary prospective population-based cohort study examining in a unique three-generation design the health and health-related behaviours of 167,729 persons living in the North of the Netherlands. It employs a broad range of investigative procedures in assessing the biomedical, socio-demographic, behavioural, physical and psychological factors which contribute to the health and disease of the general population, with a special focus on multi-morbidity and complex genetics.

Reference for study design and data collection:

1. Stolk RP, Rosmalen JGM, Postma DS, De Boer RA, Navis G, Slaets JPJ, et al. Universal risk factors for multifactorial diseases: LifeLines: A three-generation population-based study. Eur J Epidemiol. 2008;23(1):67–74.

**NEMESIS-2**

Netherlands Mental Health Survey and Incidence Study (NEMESIS) examines the prevalence, incidence, course and consequences of common mental disorders (mood, anxiety and substance use disorders) in the Dutch adult general population.   The baseline wave of NEMESIS-2 was performed between November 2007 and July 2009. Face-to-face interviews were administered with the Composite International Diagnostic Interview (CIDI) 3.0. The baseline wave of NEMESIS-2 was performed between November 2007 and July 2009. Face-to-face interviews were administered with the Composite International Diagnostic Interview (CIDI) 3.0.

Reference for study design and data collection:

1.de Graaf R, Ten Have M, van Dorsselaer S. The Netherlands Mental Health Survey and Incidence Study-2 (NEMESIS-2): design and methods. *Int J Methods Psychiatr Res* 2010; **19**: 125–41.

2. Prevalence of mental disorders and trends from 1996 to 2009. Results from the Netherlands Mental Health Survey and Incidence Study-2. Soc Psychiatry Psychiatr Epidemiol. 2012;47(2):203–13.

**PONS**

The Polish Cohort Study (PONS), formerly known as the Polish-Norwegian Study, is an ongoing population-based prospective cohort study conducted in southeastern Poland, specifically in Kielce and its surrounding rural areas. At baseline, performed in 2010–2011, the study included 13,148 individuals aged 45–64. Detailed information on the study design, recruitment procedures, and data collection has been published elsewhere. Follow-ups, including questionnaires and medical measurements, were conducted during 2018–2020 and 2023. The study was approved by the Bioethical Committee at the National Research Institute of Oncology in Poland, and all participants provided written informed consent. The Principal Investigator of the PONS study is Prof. Marta Manczuk ([marta.manczuk@nio.gov.pl](mailto:marta.manczuk@nio.gov.pl)).

Reference for study design and data collection:

Manczuk M, Boffetta P, Sartori S, Hashim D, Vatten LJ, Zatonski WA. Cohort Profile: The Polish-Norwegian Study (PONS) cohort. Int J Epidemiol. 2017 Apr 1;46(2):e5. doi: 10.1093/ije/dyv037. PMID: 25948663.

**SALIA**

The Study on the influence of Air pollution on Lung function, Inflammation and Aging (SALIA) study was performed between 1985 and 1994 as part of the Environmental Health surveys as an element of the Clean Air Plan introduced by the Government of North-Rhine Westphalia (West Germany). Initially, 4,874 women (70%) aged 55 years living between 1985 and 1994 in the urban Ruhr area and the adjacent rural Münsterland in Western Germany, were enrolled. Further details of the study have been described previously.

The study has been performed in accordance with the Declaration of Helsinki and approval was obtained from Ethics Committees of the Ruhr University, Bochum (reference number: 2732), and the Heinrich Heine University, Düsseldorf (reference number: 3507). Written informed consent from all women was received.

Reference for data collection and study design:

- Teichert, T. *et al.* (2013) ‘Association between Traffic-Related Air Pollution, Subclinical Inflammation and Impaired Glucose Metabolism: Results from the SALIA Study’, *PLOS ONE*, 8(12), p. e83042. <https://doi.org/10.1371/journal.pone.0083042>.
- Vossoughi, M., Schikowski, T., Vierkötter, A. *et al.* Air pollution and subclinical airway inflammation in the SALIA cohort study. *Immun Ageing* **11**, 5 (2014). https://doi.org/10.1186/1742-4933-11-5

# Exposure assessment

For the air pollution domain, separate land use regressions (LUR) models were built for each year from 2000 to 2019 at 25 $\times$ 25m resolution for fine particulate matter (PM_2.5_), inhalable particulate matter (PM_10_), nitrogen dioxide (NO_2_) and Ozone (O_3_) using annual average monitoring observations across Europe. Supervised linear regression was used to select the potential predictors including satellite retrievals, chemical transport model estimates and land use variables. Geographically and temporally weighted regression (GTWR) was further applied to estimate the potential spatially-varying coefficients ^1^.

For the built environment domain, greenness, grey spaces, blue spaces, artificial light at night (LAN) were assessed. Residential surrounding greenness was assessed using satellite-derived Normalized Difference Vegetation Index (NDVI) from the Terra Moderate Resolution Imaging Spectroradiometer ^2^ (MODIS) with 250 $\times$ 250 m resolution. Accessibility to green space was defined based on the distance to the nearest green space using the Corine Land Cover database^3^. Distance to the nearest inland fresh water and ocean was assessed using the EU-Hydo map developed by the Copernicus Land Monitoring service^4^. Grey spaces were assessed using the imperviousness density maps^5^. Light at night was assessed using harmonized data produced with DMSP^6^ (Defense Meteorological Satellite Program) and VIIRS^7^ (Version 1 VIIRS Day/Night Band Night-time Lights) data sources.

For the ambient temperature domain, daily mean, minimum and maximum ambient temperature surfaces with 1 $\times$1 km resolution for Europe from 2003 to 2020 were developed using a two-stage random-forest modelling approach^8^. As the current study focused on the long-term impact of urban environment, daily temperatures were assigned to the baseline addresses of participants and subsequently aggregated to calculate the annual and seasonal mean and standard deviation values (with warm season from April to September, cold season from October to March).

**Reference for exposure assessment:**

1 Shen Y, de Hoogh K, Schmitz O, *et al.* Europe-wide air pollution modeling from 2000 to 2019 using geographically weighted regression. *Environ Int* 2022; **168**: 107485.

2 NASA EOSDIS Land Processes DAAC. MOD13Q1 MODIS/Terra Vegetation Indices 16-Day L3 Global 250m SIN Grid V006. .

3 EU Hydro. Copernicus Land Monitoring Service. 2019. https://land.copernicus.eu/imagery-in-situ/eu-hydro.

4 Carroll ML, DiMiceli CM, Townshend JRG, *et al.* Development of an operational land water mask for MODIS Collection 6, and influence on downstream data products. *International Journal of Digital Earth* 2017; **10**: 207–18.

5 Copernicus Land Monitoring Service. Status Maps. 2020. https://land.copernicus.eu/pan-european/high-resolution-layers/imperviousness/status-maps.

6 Li X, Zhou Y, Zhao M, Zhao X. A harmonized global nighttime light dataset 1992–2018. *Sci Data* 2020; **7**: 168.

7 Elvidge CD, Baugh K, Zhizhin M, Hsu FC, Ghosh T. VIIRS night-time lights. *International Journal of Remote Sensing* 2017; **38**: 5860–79.

8 Bussalleu A, Hoek G, Kloog I, Probst-Hensch N, Röösli M, de Hoogh K. Modelling Europe-wide fine resolution daily ambient temperature for 2003-2020 using machine learning. *Sci Total Environ* 2024; **928**: 172454.

# Simulation for testing the cluster assignments

To test the robustness of the cluster assignment, we repeated the analysis using simulated datasets for each cohort. In detail, we first simulated the exposure of interest based on the cohort-specific summary statistics and co-variance matrix. We then performed the k-means clustering within the simulated datasets for each cohort and in the simulated pooled dataset, and calculated the weighted environmental score. We further compared the cluster assignments of the real and the simulated datasets using the adjusted rand index and compared the weighted environmental score of the real and the simulated datasets using the Pearson’s correlation index, with confidence intervals calculated based on 200 iterations


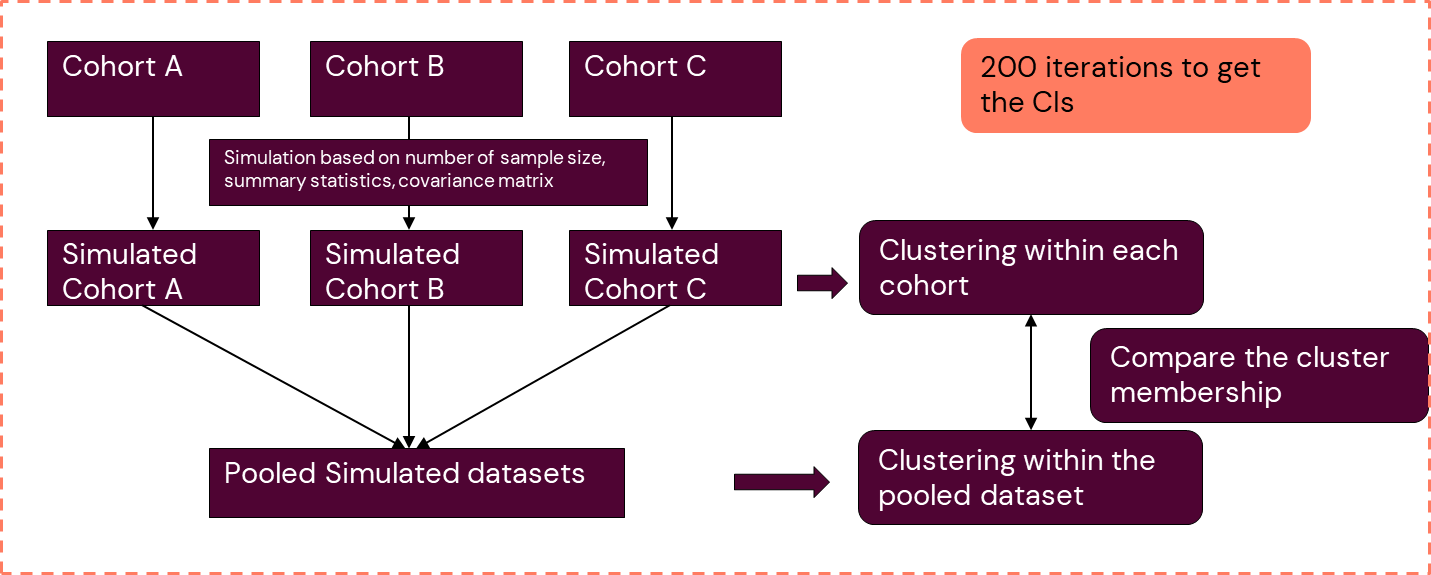


# Cohort-specific funding and acknowledgements

**BAMSE**: BAMSE cohort is supported by the Swedish Research Council (Ref no 2016-03086; 2020-01886), the Swedish Research Council for Health, Working life and Welfare (FORTE 2017-01146), the Swedish Heart-Lung Foundation and Region Stockholm (ALF and database maintenance). We thank all the children and their parents for participating in the BAMSE cohort, and the nurses and other staff members working in the BAMSE project.

**ELSPAC-CZ**: The ELSPAC-CZ study was supported by the RECETOX Research Infrastructure (No LM2023069, MEYS).

**Estonian Biobank:** The work of J.K. was supported by the Estonian Research Council grant PRG1291. The work of K.F was supported by the Estonian Research Council grant PRG1197. The work of J.K. and H.M.K leading to this article has received funding from the European Union’s Horizon 2020 research and innovation programme under grant agreement No 874627.We would like to thank the Estonian Biobank IT team for linking exposure data to biobank participants. We thank all Estonian Biobank participants.

**GCAT**:: This study utilizes data generated by the GCAT-Genomes for Life cohort study of the Genomes of Catalonia, conducted by the Fundació IGTP, which is part of the CERCA Program of the Generalitat de Catalunya. The group is supported by the Agència de Gestió d’Ajuts Universitaris i de Recerca (AGAUR) (SGR 01537), with the study has funding from the ISCIII Spanish National Grant PI18/01512.

The study was conducted using anonymized data provided by the Catalan Agency for Quality and Health Assessment within the framework of the PADRIS Program. We extend our gratitude to the volunteers who participated in the GCAT study and to all the staff of the Blood and Tissue Bank (BST). We also wish to acknowledge the GCAT project investigators for their contributions to generating the GCAT data (www.genomesforlife.com).

The Exposome-GIS data derived from the GCAT cohort, included in this study, resulted from the joint effort between ISGlobal and IGTP, in collaboration with Manolis Kogevinas (PI 17/01388). We especially thank Marta Cyrach for her technical assistance in data linkage. All GCAT codes have been linked to data generated by the EXPANSE project and deposited in the YODA repository at Utrecht University.

The authors of the study would like to acknowledge all GCAT project investigators who contributed to the generation of the GCAT data. A full list of the investigators is available from www.genomesforlife.com, specially former one, Anna Carreras. We thank Dr. Joan Grifols on behalf of the Blood and Tissue Bank from Catalonia (BST) and all the GCAT volunteers that participated in the study. This study was carried out using data provided by the Catalan Agency for Quality and Health Assessment, within the framework of the PADRIS Program.

**GINIplus LISA**: The authors thank all the families for their participation in the GINIplus and in the LISA study, and all the members of the GINIplus and LISA Study Groups for their excellent work.

The German Infant Study on the Influence of Nutrition Intervention PLUS Environmental and Genetic Influences on Allergy Development (GINIplus)

The GINIplus study was mainly supported for the first 3 years of the Federal Ministry for Education, Science, Research and Technology (interventional arm) and Helmholtz Zentrum Munich (formerlyformer GSF) (observational arm). The 4- year, 6- year, 10- year, and 15- year follow-up examinations of the GINIplus study were covered from the respective budgets of the 5 study centers (Helmholtz Zentrum Munich [formerly(former GSF],), Research Institute at Marien-Hospital Wesel, LMU Munich, TU Munich), and from year 6 onward it wasyears onwards also supported with funding from from IUF- - Leibniz Research -Institute for Environmental Medicine at the University of Düsseldorf) and by a grant from the Federal Ministry for Environment (IUF Düsseldorf [grant, FKZ 20462296]). Furthermore). Further, the 15- year follow-up examination of the GINIplus study was supported by the Commission of the European Communities, the Seventh7th Framework Program: MeDALL project, and as well by the companies Mead Johnson and Nestlé.

The Influences of Lifestyle-Related Factors on the Human Immune System and Development of Allergies in Childhood (LISA)LISA study was mainly supported by grants from the Federal Ministry for Education, Science, Research and Technology and alsoin addition from Helmholtz Zentrum Munich (formerlyformer GSF), the Helmholtz Centre for Environmental Research- - UFZ, Leipzig, the Research Institute at Marien-Hospital Wesel, Pediatric Practice, and Bad Honnef for the first 2 years. The 4- year, 6- year, 10- year, and 15- year follow-up examinations of the LISA study were covered from the respective budgets of the involved partners (Helmholtz Zentrum Munich [formerly(former GSF], the), Helmholtz Centre for Environmental Research- - UFZ, Leipzig, the Research Institute at Marien-Hospital Wesel, Pediatric Practice, Bad Honnef, and IUF– – Leibniz-Research Institute for Environmental Medicine at the University of Düsseldorf) and alsoin addition by a grant from the Federal Ministry for Environment (IUF Düsseldorf [grant, FKZ 20462296]). Furthermore). Further, the 15-year follow-up examination of the LISA study was supported by the Commission of the European Communities, the Seventh7th Framework Program: MeDALL project.

Acknowledgements:

The authors thank all the families for their participation in the GINIplus study. Furthermore, we thank all members of the GINIplus Study Group for their excellent work. The GINIplus Study group consists of the following: Institute of Epidemiology, Helmholtz Zentrum München, German Research Center for Environmental Health, Neuherberg (Heinrich J, Brüske I, Schulz H, Flexeder C, Zeller C, Standl M, Schnappinger M, Ferland M, Thiering E, Tiesler C); Department of Pediatrics, Marien-Hospital, Wesel (Berdel D, von Berg A); Ludwig-Maximilians-University of Munich, Dr von Hauner Children’s Hospital (Koletzko S); Child and Adolescent Medicine, University Hospital rechts der Isar of the Technical University Munich (Bauer CP, Hoffmann U); IUF- Environmental Health Research Institute, Düsseldorf (Schikowski T, Link E, Klümper C, Krämer U, Sugiri D).

The authors thank all the families for their participation in the LISA study. Furthermore, we thank all members of the LISA Study Group for their excellent work. The LISA Study group consists of the following: Helmholtz Zentrum München, German Research Center for Environmental Health, Institute of Epidemiology, Munich (Heinrich J, Schnappinger M, Brüske I, Ferland M, Schulz H, Zeller C, Standl M, Thiering E, Tiesler C, Flexeder C); Department of Pediatrics, Municipal Hospital “St. Georg”, Leipzig (Borte M, Diez U, Dorn C, Braun E); Marien Hospital Wesel, Department of Pediatrics, Wesel (von Berg A, Berdel D, Stiers G, Maas B); Pediatric Practice, Bad Honnef (Schaaf B); Helmholtz Centre of Environmental Research – UFZ, Department of Environmental Immunology/Core Facility Studies, Leipzig (Lehmann I, Bauer M, Röder S, Schilde M, Nowak M, Herberth G , Müller J); Technical University Munich, Department of Pediatrics, Munich (Hoffmann U, Paschke M, Marra S); Clinical Research Group Molecular Dermatology, Department of Dermatology and Allergy, Technische Universität München (TUM), Munich (Ollert M, J. Grosch).

**PONS**: Establishing the Polish Cohort Study PONS was co-finances from the Polish-Norwegian Research Fund (PNRF-228-AI-1/07).

**PIAMA:**

The PIAMA study was supported by The Netherlands Organization for Health Research and Development; The Netherlands Organization for Scientific Research; Lung Foundation Netherlands (Longfonds); The Netherlands Ministry of Spatial Planning, Housing, and the Environment; The Netherlands Ministry of Health, Welfare, and Sport; and the National Institute for Public Health and the Environment (RIVM).

**SALIA:**

Funding:

The IUF is funded by the federal and state governments - the Ministry of Culture and Science of North Rhine-Westphalia (MKW) and the Federal Ministry of Education and Research (BMBF).

The SALIA cohort study was supported by grants from the Deutsche Forschungsgemeinschaft (DFG) [HE-4510/2-1, KR 1938/3-1, LU 691/4-1 and SCHI 1358/3-1], the Ministry of the Environment of the state North Rhine-Westphalia (Düsseldorf, Germany), the Federal Ministry of the Environment (Berlin, Germany), the German Statutory Accident Insurance (DGUV) [VT 266.1], the European Research Council (ERCCommunity’s Seventh Framework Program (FP7/2007-2011) under the Europeangrant agreement number [211250] and the German Federal Ministry of Education and Research (BMBF). Union’s Horizon 2020 research and innovation programme (grant agreement No. 949906).

Acknowledgements:

SALIA group: Study directorate: R Dolgner; U Krämer, U Ranft, T Schikowski, A Vierkötter. Scientific Team Baseline: AW Schlipköter, MS Islam; A Brockhaus, H Idel, R Stiller-Winkler, W Hadnagy, T Eikmann, Scientific Team Follow-up: D Sugiri, A Hüls, B Pesch, A Hartwig, H Käfferlein, V Harth, T Brüning, T Weiss. Study Nurses: G Seitner-Sorge, V Jäger, G Petczelies, I Podolski, T Hering, M Goseberg. Administrative Team: B Schulten, S Stolz.

We thank all study members and staff involved in data collection and also the respective funding bodies. During the last decades, many scientists, study nurses and laboratories were involved in conducting the studies. We are most grateful for all the individuals who participated in the study over decades.

**HAPIEE**:

The baseline survey was funded by Wellcome Trust and McArthur Foundation. The follow-up investigation in the Czech Republic was conducted in 2023-2024 and was supported by the NPO „Systemic Risk Institute“ (LX22NPO5101), funded by European Union – Next Generation EU (Ministry of Education, Youth and Sports, NPO: EXCELES). The follow-up investigation was supported from the European Union’s Horizon 2020 research and innovation programme under grant agreement No 857487 (R-Exposome Chair), No 857560 (CETOCOEN Excellence). Authors thank the RECETOX Research Infrastructure (No LM2023069) financed by the Ministry of Education, Youth and Sports for supportive background.

# Supplemental Table 1 Asthma definitions in the cohorts

| Cohort type | Cohort name | Ascertainment of asthma cases | Detailed definition |
| --- | --- | --- | --- |
| Mature birth cohorts | BAMSE | Questionnaire, MeDALL definition^1^ | The presence of at least two of the following three criteria: 1) doctor-diagnosed asthma ever; 2) symptoms of wheeze and/or breathing difficulties in the last 12 months prior to the date of questionnaire; 3) use of any asthma medication occasionally or regularly in the last 12 months prior to the date of questionnaire. |
|  | GINIplusLISA_south | Questionnaire, MeDALL definition |  |
|  | GINIplusLISA_north | Questionnaire, MeDALL definition |  |
|  | PIAMA | Questionnaire MeDALL definition |  |
|  | Krakow | Questionnaire | Positive answer to the doctor-diagnosed asthma during the follow-up questionnaire |
|  | ELSPAC-CZ | Linkage to electronic health records based on ICD codes | Paediatric health records; children who had a record of paediatrician-diagnosed asthma (coded as J45 or J46 in International Classification of Diseases (ICD)-10, or 493.0, 493.1 or 493.9 in ICD-9) treated by medications |
| Adult cohorts | CEANS | Linkage to the electronic health records based on ICD codes | ICD-9 490-492 or ICD-10 J40-J44 |
|  | EstBB_1 and EstBB_2 | Linkage to the electronic health records based on ICD-10 codes | We used both electronic health records (EHR) and self-reported diagnoses. Self-reported diagnoses were only used for the definition of prevalent disease and excluded form both cases and controls.  Cases were defined based on EHR: J45 or J46 at least once AND a medication prescribed for the diagnosis of J45 or J46. Participants with only diagnosis or only prescription were excluded from cases and controls. People with no diagnoses in electronic health records were excluded (as they are likely not living in Estonia or using the healthcare system). COPD diagnoses were excluded from cases and controls (J40-J44). Cases were further divided into prevalent and incident, with baseline set to 2014 for the EstBB_1 and 2018 for the EstBB_2. |
|  | GCAT | Linkage to the electronic health records based on ICD codes | ICD-9 490-492 or ICD-10 J40-J44 |
|  | HAPIEE | Questionnaire | Positive answer to the doctor-diagnosed asthma during the follow-up questionnaire |
|  | Lifelines | Questionnaire, MeDALL definition | See above MeDALL definition |
|  | NEMESIS-2 | Questionnaire | Positive answer to the doctor-diagnosed asthma during the follow-up questionnaire |
|  | PONS | Linkage to the electronic health records based on ICD codes | ICD-9 490-492 or ICD-10 J40-J44 |
|  | SALIA | Questionnaire, MeDALL definition | See above for MeDALL definition |

^1^:Reference for the MeDALL definition:

Pinart, M. *et al.* Comorbidity of eczema, rhinitis, and asthma in IgE-sensitised and non-IgE-sensitised children in MeDALL: a population-based cohort study. *Lancet Respir Med* **2**, 131–40 (2014).

# Supplemental Table 2 Information for adjusted covariates and baseline year in each cohort

| Cohort | Baseline year of the cohort | Index year for air pollution exposure | Index year for built environment | Index year for temperature | Covariates adjusted |
| --- | --- | --- | --- | --- | --- |
| BAMSE | 1994-1996 | 2000 | 2000 for NDVI, LAN; 2013 for BIS_DIS_DIS, BSS_DIS_DIS, 2006 for GSC, IMP | 2003 | age, sex, parental education, parental asthma/hay fever, breastfeeding, native nationality, day care attendance, older siblings, maternal smoking, environmental tobacco smoking, mould/dampness at home, pets, use of gas cooking, active smoking |
| PIAMA | 1996-1997 | 2000 |  | 2003 |  |
| GINIplusLISA south | 1995-1999 | 2000 |  | 2003 |  |
| GINIplusLISA north | 1995-1999 | 2000 |  | 2003 |  |
| Krakow | 2000-2004 | 2000-2003 |  | 2003 |  |
| ELSPAC_CZ | 1991-1992 | 2000 |  | 2003 |  |
| CEANS | 1992-2004 | 2000-2003 | 2000 for NDVI, LAN; 2013 for BIS_DIS_DIS, BSS_DIS_DIS, 2006 for GSC, IMP | 2003 | age, sex, smoking status, BMI, marital status, employment status, education level, area-level SES |
| EstBB_1 | 2013 | 2013 | 2015 for NDVI, IMP LAN, 2012 for GSC,2013 for BIS_DIS_DIS, BSS_DIS_DIS | Not applicable | age, sex, smoking status, BMI, employment status, education level, area-level SES |
| EstBB_2 | 2018 | 2018 | 2018 for NDVI, IMP, LAN; 2013 for BIS_DIS, BSS_DIS, 2018 for GSC | 2018 | age, sex, smoking status, BMI, employment status, education level |
| GCAT | 2014-2018 | 2013-2017 | 2015/2018 fr NDVI, IMP,LAN, 2018 for GSC, 2013 for BIS_DIS_DIS, BSS_DIS_DIS, | 2013-2017 | age, sex, smoking status, BMI, marital status, employment status, education level, area-level SES |
| HAPIEE | 2002-2005 |  | 2000/2005 for NDVI, IMP, LAN; 2013 for BIS_DIS, BSS_DIS, 2006 for GSC | 2003-2004 | age, sex, smoking status, BMI, marital status, employment status, education level |
| Lifelines | 2006-2014 | 2005-2013 | 2005, 2010 for NDVI,2006, 2009, 2012 for IMP, 2013 for BIS_DIS, BSS_DIS, 2006/2012 for GSC | 2005-2013 | age, sex, smoking status, BMI, marital status, employment status, education level, area-level SES |
| NEMESIS-2 | 2007-2009 | 2006-2008 | 2005 for NDVI, LAN; 2006 for IMP, GSC; 2013 for BIS_DIS, BSS_DIS | 2006-2008 | age, sex, smoking status, BMI, marital status, employment status, education level, area-level SES |
| PONS | 2010-2012 | 2009-2011 | 2010 for NDVI, LAN, 2012 for IMP, GSC, 2013 for BIS_DIS, BSS_DIS | 2009-2011 | age, sex, smoking status, BMI, marital status, employment status, education level, area-level SES |
| SALIA | 1985-1994 | 2000 | 2000 for NDVI, IMP, LAN; 2006 for BIS_DIS, BSS_DIS, GSC | 2003 | age, sex, smoking status, BMI, marital status, employment status, education level, area-level SES |

# Supplemental Table 3 Associations between the urban environment clusters with asthma incidence across the cohorts

| Cohort type | Cohorts | Air pollution domain (clean air with elevated O_3_ as reference) | | | | Built environment domain (high greenness-low built-up cluster as reference) | | | | Ambient temperature domain (Low annual temperature as reference) | | | |
| --- | --- | --- | --- | --- | --- | --- | --- | --- | --- | --- | --- | --- | --- |
|  |  | Moderate pollution cluster | | High PM-NO2- but low O3 cluster | | Moderate built environment cluster | | Low greenness-high built up clusters | | Moderate annual temperature | | High annual temperature | |
|  |  | OR (95%CI) | P value | OR (95%CI) | P value | OR (95%CI) | P value | OR (95%CI) | P value | OR (95%CI) | P value | OR (95%CI) | P value |
| Mature birth cohorts^a^ | BAMSE | 1.13 (0.91, 1.41) | 0.274 | 1.06 (0.80,1.41) | 0.687 | 1.06 (0.85, 1.31) | 0.597 | 1.24 (0.93, 1.65) | 0.141 | 0.96 (0.82, 1.14) | 0.627 | 0.79 (0.42, 1.48) | 0.463 |
|  | ELSPAC-CZ | 1.05 (0.71, 1.56) | 0.808 | 1.27 (0.86,1.86) | 0.224 | 1.07 (0.70, 1.64) | 0.755 | 1.66 (1.10, 2.49) | 0.015 | 0.82 (0.52, 1.31) | 0.400 | 0.59 (0.38, 0.90) | 0.016 |
|  | GINIplus/LISA North | 1.37 (0.58, 3.22) | 0.472 | 1.55 (0.80, 3.00) | 0.194 | 1.64 (0.85, 3.15) | 0.139 | 1.63 (0.93, 2.86) | 0.088 | 1.17 (0.57, 2.39) | 0.668 | 0.86 (0.46, 1.62) | 0.639 |
|  | GINIplus/LISASouth | 0.95 (0.58, 1.53) | 0.835 | 0.92 (0.47,1.81) | 0.808 | 0.97 (0.66, 1.43) | 0.877 | 1.32 (0.73, 2.39) | 0.359 | 1.03 (0.63, 1.67) | 0.905 | 0.99 (0.52, 1.87) | 0.975 |
|  | Krakow | 1.57 (0.57, 4.27) | 0.380 | 1.95 (0.90, 4.19) | 0.089 | 1.08 (0.47, 2.50) | 0.857 | 1.02 (0.52, 2.00) | 0.954 | 1.38 (0.65, 2.93) | 0.402 | 1.28 (0.60, 2.73) | 0.523 |
|  | PIAMA | 1.24 (0.73, 2.11) | 0.427 | 1.11 (0.60, 2.07) | 0.741 | 1.45 (1.05, 2.00) | 0.024 | 1.45 (0.82, 2.56) | 0.201 | 1.12 (0.62, 2.01) | 0.706 | 1.22 (0.75, 2.01) | 0.429 |
|  | Meta-analysis^c^ | 1.12 (0.95, 1.31) | 0.167 | 1.18 (0.97, 1.42) | 0.089 | 1.14 (0.98, 1.34) | 0.101 | 1.36 (1.14, 1.64) | 0.0009 | 0.98 (0.86, 1.13) | 0.772 | 0.89 (0.67, 1.17) | 0.413 |
|  |  |  |  |  |  |  |  |  |  |  |  |  |  |
| Adult cohorts^b^ | CEANS | 1.58 (1.16, 2.16) | 0.004 | 1.30 (0.86, 1.98) | 0.217 | 1.17 (0.76, 1.81) | 0.478 | 1.20 (0.68, 2.12) | 0.530 | 0.91 (0.64, 1.29) | 0.598 | 1.47 (1.01, 2.16) | 0.047 |
|  | EstBB_1 | 0.87 (0.69, 1.10) | 0.242 | 1.05 (0.83, 1.32) | 0.680 | 1.42 (1.04, 1.94) | 0.027 | 1.19 (0.96, 1.48) | 0.115 | 1.12 (0.91, 1.39) | 0.294 | 1.07 (0.81, 1.40) | 0.628 |
|  | EstBB_2 | 0.94 (0.75, 1.18) | 0.593 | 1.31 (1.01, 1.70) | 0.042 | 1.45 (1.11, 1.90) | 0.007 | 1.24 (0.97, 1.57) | 0.080 | - |  | - | - |
|  | GCAT | 0.98 (0.64, 1.51) | 0.926 | 0.83 (0.53, 1.31) | 0.420 | 1.18 (0.85, 1.65) | 0.328 | 0.77 (0.41, 1.45) | 0.417 | 0.72 (0.39, 1.33) | 0.293 | 0.80 (0.54, 1.18) | 0.263 |
|  | HAPIEE | 0.83 (0.43, 1.59) | 0.576 | 1.79 (0.98, 3.26) | 0.058 | 1.52 (0.75, 3.08) | 0.245 | 2.46 (1.00, 6.04) | 0.049 | 1.22 (0.42, 3.56) | 0.715 | 2.33 (0.79, 6.90) | 0.126 |
|  | Lifelines | 1.07 (0.95, 1.21) | 0.273 | 1.13 (0.96, 1.34) | 0.151 | 1.21 (0.97, 1.47) | 0.072 | 1.12 (0.95, 1.35) | 0.206 | 1.12 (0.96, 1.30) | 0.143 | 0.99 (0.88, 1.12) | 0.870 |
|  | NEMESIS | 0.82 (0.47, 1.42) | 0.482 | 0.79 (0.40, 1.53) | 0.491 | 1.02 (0.64, 1.61) | 0.933 | 0.85 (0.44, 1.64) | 0.628 | 1.21 (0.79, 1.87) | 0.386 | 0.85 (0.44, 1.63) | 0.627 |
|  | PONS | 0.54 (0.27, 1.09) | 0.083 | 0.73 (0.33, 1.64) | 0.442 | 1.05 (1.00, 1.10) | 0.045 | 0.69 (0.30, 1.55) | 0.376 | 1.16 (0.54, 2.50) | 0.704 | 1.19 (0.52, 2.73) | 0.681 |
|  | SALIA | 1.55 (0.54, 4.48) | 0.417 | 0.75 (0.24, 2.41) | 0.625 | 1.21 (0.40, 3.63) | 0.735 | 1.99 (0.62, 6.46) | 0.250 | 1.52 (0.51, 4.50) | 0.451 | 0.54 (0.17, 1.67) | 0.290 |
|  | Meta-analysis^c^ | 1.00 (0.85, 1.18) | 1 | 1.13 (1.01, 1.25) | 0.025 | 1.19 (1.05, 1.34) | 0.005 | 1.15 (1.03, 1.28) | 0.012 | 1.09 (0.98, 1.22) | 0.123 | 1.02 (0.92, 1.13) | 0.706 |

^a^: Estimates in the birth cohorts were adjusted depending on the availability in the cohorts for age (dummy variable), sex, parental education, parental asthma/hay fever, breastfeeding, native nationality, day care attendance, older siblings, maternal smoking, environmental tobacco smoking, mould/dampness at home, pets, use of gas cooking, active smoking and mutually adjusted for the other two environmental domains.

^b^: Estimates in the adult cohorts were adjusted depending on the availability in the cohorts for age, sex, smoking status, BMI, marital status, employment status, education level, area-level SES and mutually adjusted for the other two environmental domains. For HAPIEE, results from single-environment domain were used as multicollinearity happened for tor multi-domain model.

^C^: Meta-analysis was conducted using random-effects model.

# Supplemental Table 4 Associations between unweighted environmental score with asthma incidence across cohorts

| Cohort type | Cohort | OR (95%CI) | P-value | Weight (%) | I^2^ |
| --- | --- | --- | --- | --- | --- |
| Mature birth cohorts (MBC) | BAMSE | 1.09 (0.95, 1.25) | 0.236 | 7.8 | - |
|  | GINIplusLISA south | 1.11 (0.93, 1.33) | 0.226 | 5.7 | - |
|  | GINIplusLISA north | 1.42 (1.02, 1.99) | 0.187 | 2.0 | - |
|  | PIAMA | 1.24 (1.10, 1.41) | <0.001 | 8.5 | - |
|  | Krakow | 1.31 (0.92, 1.85) | 0.003 | 1.9 | - |
|  | ELSPAC_CZ | 1.42 (1.13, 1.80) | 0.003 | 3.7 | - |
|  | **Meta-analysis (MBC)** | **1.21 (1.10, 1.32)** | **<0.0001** | **-** | **19.8%** |
| Adult cohorts (AC) | CEANS | 1.24 (1.04, 1.49) | 0.018 | 5.5 | - |
|  | EstBB_2 | 1.07 (0.98, 1.18) | 0.149 | 8.6 | - |
|  | EstBB_1 | 1.06 (0.93, 1.20) | 0.402 | 11.1 | - |
|  | GCAT | 1.20 (0.91, 1.57) | 0.197 | 2.9 | - |
|  | HAPIEE | 1.02 (0.99, 1.04) | 0.054 | 17.6 | - |
|  | Lifelines | 1.05 (1.01, 1.10) | 0.009 | 16.2 | - |
|  | NEMESIS-2 | 1.05 (0.81, 1.35) | 0.729 | 3.2 | - |
|  | PONS | 1.06 (0.87, 1.30) | 0.549 | 4.6 | - |
|  | SALIA | 1.47 (0.78, 2.75) | 0.530 | 0.6 | - |
|  | **Meta-analysis (AC)** | **1.05 (1.02, 1.09)** | **<0.0001** | **-** | **18.8%** |

Estimates in the birth cohorts were adjusted depending on the availability in the cohorts for age (dummy variable), sex, parental education, parental asthma/hay fever, breastfeeding, native nationality, day care attendance, older siblings, maternal smoking, environmental tobacco smoking, mould/dampness at home, pets, use of gas cooking, active smoking and in the adult cohorts were adjusted depending on the availability in the cohorts for age, sex, smoking status, BMI, marital status, employment status, education level, area-level SES.

# Supplemental Table 5 Percentage of missing category in the covariates across cohort and associations between environmental risk score with asthma incidence across cohorts using the complete-set dataset

| **Mature birth cohorts** | **BAMSE** | | **ELSPAC_CZ** | | | **GINILISA_south** | | **GINILISA_north** | | **Krakow** | | **PIAMA** | |
| --- | --- | --- | --- | --- | --- | --- | --- | --- | --- | --- | --- | --- | --- |
| N | 3792 | | 5151 | | | 2889 | | 1969 | | 413 | | 3687 | |
| parental education | 2 (0.1%) | | 690 (13.4%) | | | 10 (0.3%) | | 6 (0.3%) | | 0 | | 31 (0.8%) | |
| parental asthma | 0 | | 261 (5.1%) | | | 57 (2.0%) | | 11 (0.6%) | | 47 (11.4%) | | 19 (0.5%) | |
| breastfeeding | 500 (13.2%) | | 506 (9.8%) | | | 104 (3.6%) | | 36 (1.8%) | | 0 | | 107 (2.9%) | |
| daycare attendance | 0 | | 1149 (22.3%) | | | 397 (13.7%) | | 16 (0.8%) | | 0 | | 90 (2.4%) | |
| old siblings | 0 | | 206 (4.0%) | | | 3 (0.1%) | | 6 (0.3%) | | 0 | | 8 (2.2%) | |
| environmental tobacco smoking | 19 (0.5%) | | 526 (10.2%) | | | 193 (6.7%) | | 15 (0.8%) | | 0 | | 1 (0.02%) | |
| maternal smoking during pregnancy | 1 (0.02%) | | 513 (10.0%) | | | 246 (8.5%) | | 28 (1.4%) | | 0 | | 35 (0.9%) | |
| mould and dampness | 47 (1.2%) | | 549 (10.7%) | | | 127 (4.4%) | | 41 (2.1%) | | 0 | | 22 (0.6%) | |
| pets | 33 (0.9%) | | 495 (9.6%) | | | 13 (0.5%) | | 15 (0.8%) | | 0 | | 19 (0.5%) | |
| gas cooking | 33 (0.9%) | | 522 (10.1%) | | | 18 (0.6%) | | 17 (0.9%) | | 1 (0.2%) | | 13 (0.4%) | |
| Complete case (%) | 3279 (86.5%) | | 3729 (72.4%) | | | 1721 (59.6%) | | 1865 (94.7%) | | 366 (88.6%) | | 3502 (95.0%) | |
| Estimates in the main analysis | 1.11 (1.03, 1.20) | | 1.29 (1.11, 1.50) | | | 1.09 (0.99, 1.20) | | 1.28 (1.04, 1.58) | | 1.25 (1.00, 1.53) | | 1.17 (1.07, 1.28) | |
| Estimates using the complete set | 1.14 (1.04, 1.22) | | 1.28 (1.10, 1.49) | | | 1.09 (0.96, 1.24) | | 1.15 (1.01, 1.31) | | 1.28 (1.10, 1.49) | | 1.17 (1.07, 1.28) | |
| **Adult cohorts** | **CEANS** | **EstBB_1** | | **EstBB_2** | **GCAT** | | **HAPIEE** | **Lifelines** | **NEMESIS-2** | | **PONS** | | **SALIA** |
| N | 19357 | 37890 | | 122408 | 15604 | | 1111 | 116578 | 4796 | | 12329 | | 1063 |
| Smoking status | 337 (1.7%) | 0 | | 0 | 0 | | 5 (0.5%) | 3 (0.0%) | 0 | | 7 (0.1%) | | 5 (0.5%) |
| Married status | 241 (1.2%) | 0 | | 0 | 0 | | 3 (0.3%) | 32 (0.0%) | 0 | | 11 (0.1%) | | 9 (0.8%) |
| Employment | 239 (1.3%) | 0 | | 0 | 0 | | 6 (0.5%) | 318 (0.3%) | 0 | | 0 | | 0 |
| Education level | 538 (2.8%) | 0 | | 0 | 0 | | 1 (0.09%) | 1272 (1.1%) | 0 | | 22 (0.2%) | | 7 (0.7%) |
| Area-level SES | 66 (0.3%) | 0 | | 0 | 0 | | 0 | 26 (0.0%) | 0 | | 0 | | 115 (10.8%) |
| Complete case (%) | 18580 (96.0%) | 100% | | 100% | 100% | | 993 (89.4%) | 115040 (98.7%) | 100% | | 11188 (99.8%) | | 973 (91.5%) |
| Estimates in the main analysis | 1.18 (1.07, 1.31) | 1.15 (1.04, 1.27) | | 1.15 (1.04, 1.27) | 1.08 (0.93, 1.24) | | 1.08 (0.93, 1.24) | 1.06 (1.04, 1.08) | 1.17 (0.97, 1.41) | | 1.15 (1.00, 1.33) | | 1.24 (0.98, 1.57) |
| Estimates using the complete set | 1.18 (1.07, 1.30) | 1.15 (1.04, 1.27) | | 1.15 (1.04, 1.27) | 1.08 (0.93, 1.24) | | 1.22 (1.09, 1.37) | 1.06 (1.04, 1.08) | 1.17 (0.91, 1.41) | | 1.15 (1.00, 1.33) | | 1.26 (0.95, 1.65) |

Estimates in the birth cohorts were adjusted depending on the availability in the cohorts for age (dummy variable), sex, parental education, parental asthma/hay fever, breastfeeding, native nationality, day care attendance, older siblings, maternal smoking, environmental tobacco smoking, mould/dampness at home, pets, use of gas cooking, active smoking and in the adult cohorts were adjusted depending on the availability in the cohorts for age, sex, smoking status, BMI, marital status, employment status, education level, area-level SES.

# Supplemental Table 6 Associations between environmental risk score with asthma incidence in SALIA and Lifelines additionally adjusted for indoor environmental exposure

| Cohorts | Odds ratios (95%CI) for the environmental risk score in main analysis | P value | Odds ratios (95%CI) for the environmental risk score additionally adjusted for indoor environmental exposures | P value |
| --- | --- | --- | --- | --- |
| Lifelines | 1.06 (1.04, 1.08) | 0.002 | 1.06 (1.03, 1.09) | 0.002 |
| SALIA | 1.24 (0.98, 1.57) | 0.035 | 1.23 (0.99, 1.54) | 0.066 |

Estimates in the both cohorts were adjusted depending on the availability in the cohorts for age (dummy variable), sex, smoking status, BMI, marital status, employment status, education level, area-level SES.

**Supplemental Table 7** Associations between environmental risk score with asthma incidence stratified by age 4 years in the mature birth cohorts

| Cohorts | Age Strata | Number of observations | Odds Ratio (95%CI) | P value |
| --- | --- | --- | --- | --- |
| BAMSE | Age $\leq$ 4 years | 7070 | 1.21 (1.08, 1.35) | 0.001 |
|  | Age $>$ 4 years | 12267 | 1.07 (1.01, 1.14) | 0.024 |
| ELSPAC_CZ | Age $\leq$ 4 years | 10238 | 1.28 (0.60, 2.74) | 0.527 |
|  | Age $>$ 4 years | 16124 | 1.27 (0.80, 2.03) | 0.310 |
| Krakow | Age $\leq$ 4 years | 411 | 1.34 (1.02, 1.76) | 0.034 |
|  | Age $>$ 4 years | 2097 | 1.46 (1.10, 1.96) | 0.010 |
| PIAMA | Age $\leq$ 4 years | 14817 | 1.05 (1.01, 1.12) | 0-026 |
|  | Age $>$ 4 years | 25976 | 1.27 (1.08, 1.50) | 0.004 |

Estimates in the birth cohorts were adjusted depending on the availability in the cohorts for age (dummy variable), sex, parental education, parental asthma/hay fever, breastfeeding, native nationality, day care attendance, older siblings, maternal smoking, environmental tobacco smoking, mould/dampness at home, pets, use of gas cooking, active smoking.

In the other two cohorts (GINIplusLISA north and south), asthma diagnosis under age 4 was not included in the data.

# Supplemental Table 8 Comparison of the association between environmental risk score and asthma incidence using MeDALL definition and doctor diagnosis only

| Cohort | Asthma based on MeDALL definition | | Asthma based on doctor diagnosis | |
| --- | --- | --- | --- | --- |
|  | OR (95%CI) | P value | OR (95%CI) | P value |
| BAMSE | 1.07 (1.01, 1.14) | 0.016 | 1.13 (1.04, 1.22) | 0.004 |
| GINIplusLISA_north | 1.28 (1.04, 1.58) | 0.019 | 1.18 (1.04, 1.35) | 0.013 |
| GINIplusLISA_south | 1.09 (0.99, 1.20) | 0.071 | 1.16 (1.04, 1.29) | 0.008 |
| PIAMA | 1.17 (1.07, 1.28) | 0.002 | 1.05 (0.96, 1.16) | 0.279 |

Estimates in the birth cohorts were adjusted depending on the availability in the cohorts for age (dummy variable), sex, parental education, parental asthma/hay fever, breastfeeding, native nationality, day care attendance, older siblings, maternal smoking, environmental tobacco smoking, mould/dampness at home, pets, use of gas cooking, active smoking.

# Supplemental Table 9 Leave-one-out meta-analysis for the association between environmental risk scores with asthma incidence

| Cohort type | Cohort left out | OR_meta_ (95%CI) | P value |
| --- | --- | --- | --- |
| Mature birth cohorts | No left out | 1.15 (1.08, 1.23) | <0.0001 |
|  | BAMSE | 1.18 (1.10, 1.26) | <0.0001 |
|  | GINIplusLISA north | 1.14 (1.07, 1.21) | <0.0001 |
|  | GINIplusLISA south | 1.18 (1.08, 1.28) | <0.0001 |
|  | PIAMA | 1.16 (1.06, 1.26) | <0.0001 |
|  | Krakow | 1.15 (1.07, 1.23) | <0.0001 |
|  | ELSPAC_CZ | 1.12 (1.06, 1.19) | <0.0001 |
| Adult cohorts | No left out | 1.13 (1.08, 1.18) | <0.0001 |
|  | CEANS | 1.12 (1.07, 1.17) | <0.0001 |
|  | EstBB_1 | 1.13 (1.07, 1.18) | <0.0001 |
|  | EstBB_2 | 1.13 (1.07, 1.18) | <0.0001 |
|  | GCAT | 1.13 (1.08, 1.19) | <0.0001 |
|  | HAPIEE | 1.12 (1.07, 1.17) | <0.0001 |
|  | Lifelines | 1.16 (1.11, 1.21) | <0.0001 |
|  | NEMESIS-2 | 1.13 (1.08, 1.19) | <0.0001 |
|  | PONS | 1.13 (1.08, 1.18) | <0.0001 |
|  | SALIA | 1.12 (1.07, 1.16) | <0.0001 |

# Supplemental Figure 1 Correlations between urban environmental exposures at baseline addresses


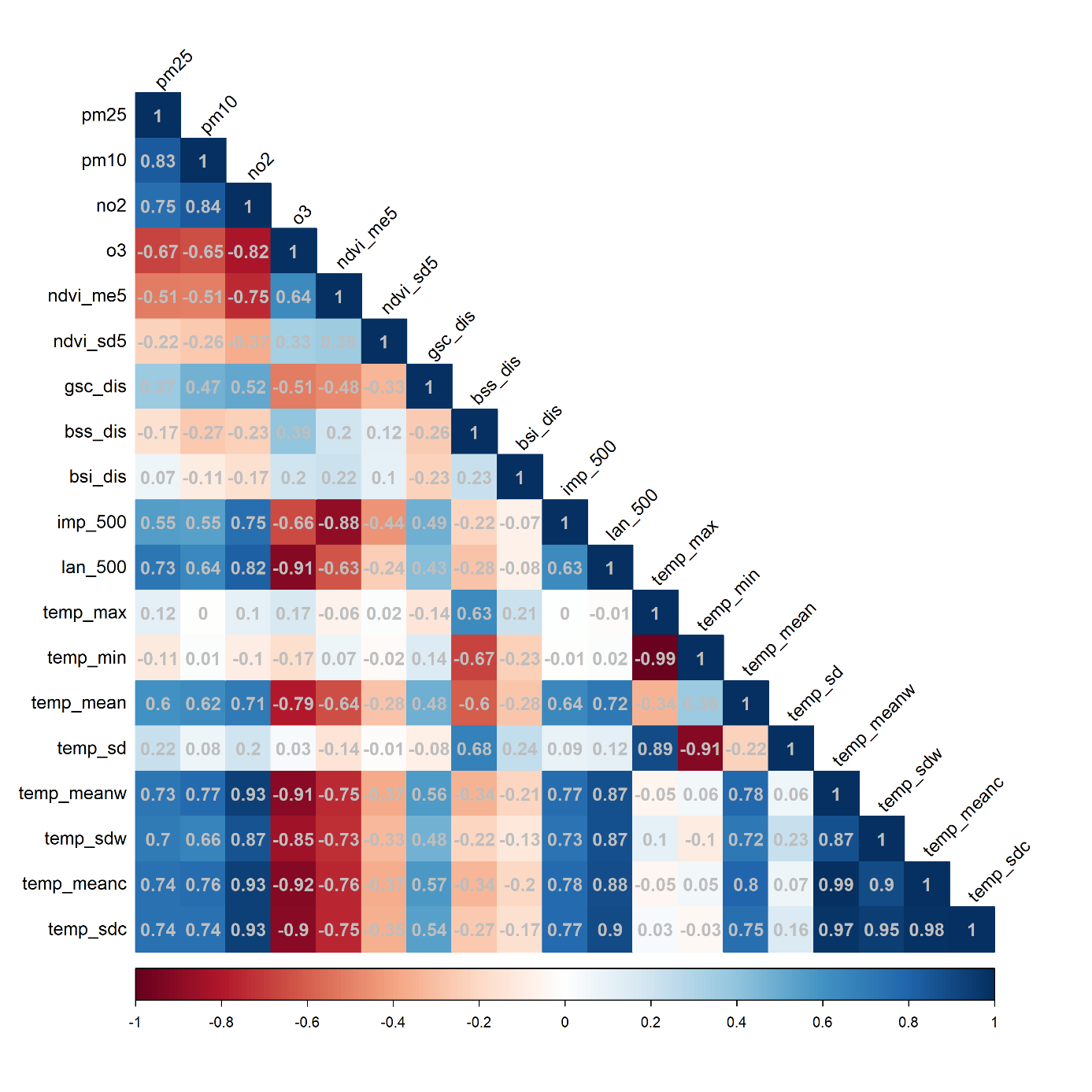


Spearman correlations were presented in the figure. Example correlation figure from the CEANS cohort. Results for all cohorts can be found at the online repository: <https://github.com/kevininef/external_exposome_asthma>

# Supplemental Figure 2 Meta-analysis of single exposure models with asthma incidence in the air pollution domain


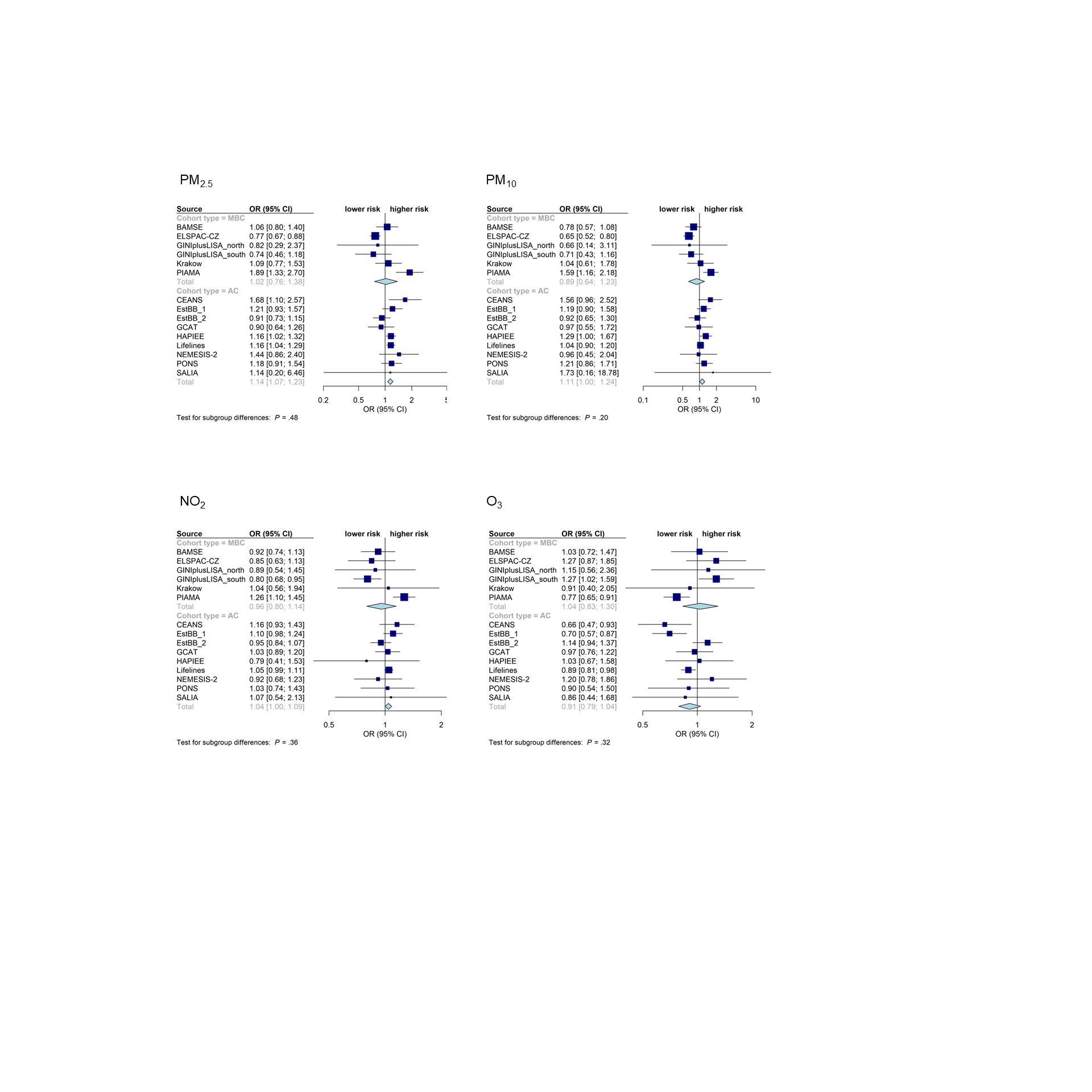


Estimates in the birth cohorts were adjusted depending on the availability in the cohorts for age (dummy variable), sex, parental education, parental asthma/hay fever, breastfeeding, native nationality, day care attendance, older siblings, maternal smoking, environmental tobacco smoking, mould dampness at home, pets, use of gas cooking, active smoking and in the adult cohorts were adjusted depending on the availability in the cohorts for age, sex, smoking status, BMI, marital status, employment, education level, area-level SES.

Odds ratios are presented per 5 μg/m^3^ increase for PM_2.5_, per 10 μg/m^3^ increase for PM_10_, NO_2_ and O_3_.

Cohort-specific and meta-analysis results in table format can be found in **Supplemental Table 10**.

# Supplemental Figure 3 Meta-analysis of single exposure models with asthma incidence in the built environment domain


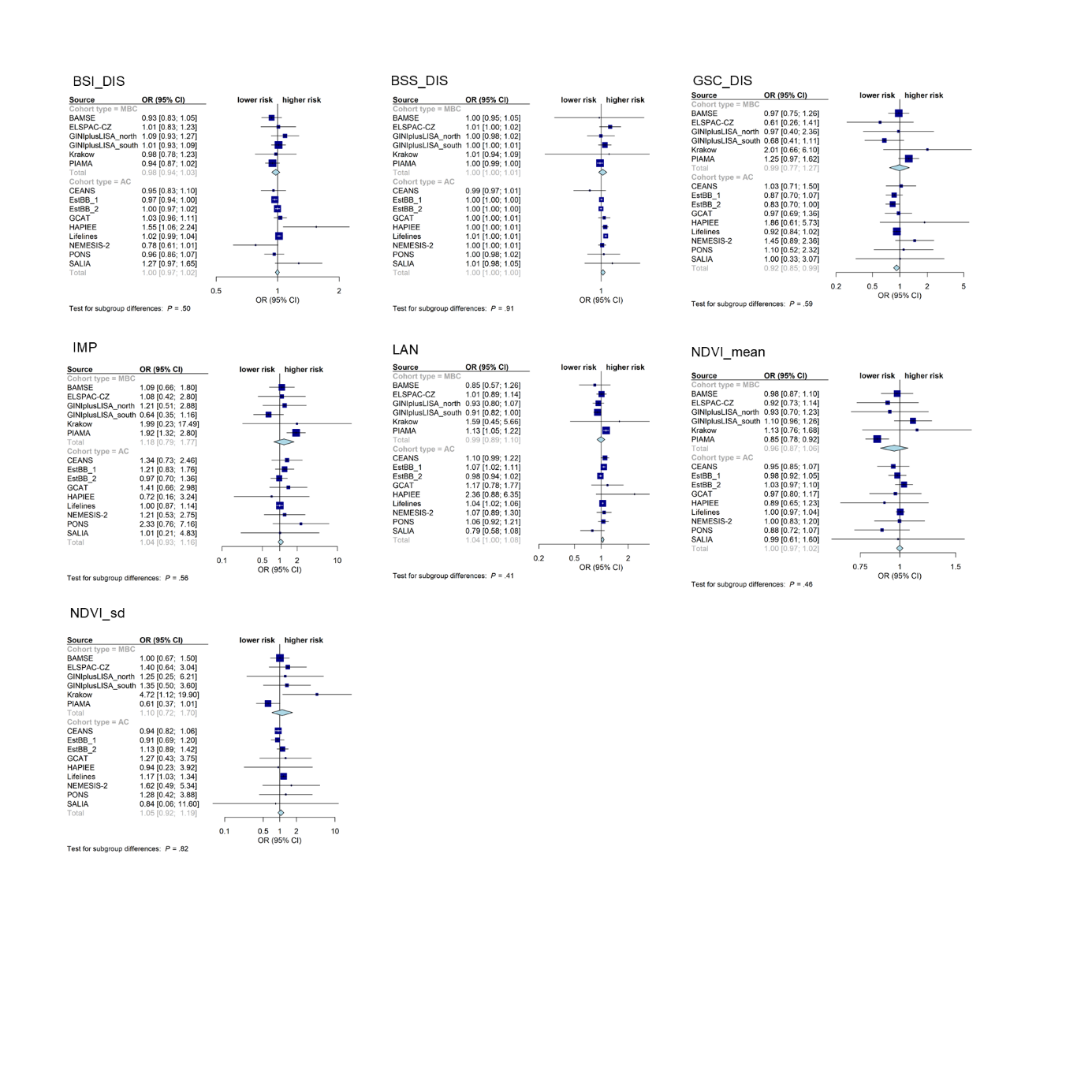


Estimates in the birth cohorts were adjusted- depending on the availability in the cohorts for age (dummy variable), sex, parental education, parental asthma/hay fever, breastfeeding, native nationality, day care attendance, older siblings, maternal smoking, environmental tobacco smoking, mould dampness at home, pets, use of gas cooking, active smoking and in the adult cohorts were adjusted depending on the availability in the cohorts for age, sex, smoking status, BMI, marital status, employment, education level, area-level SES.

Odds ratios are presented per 0.1 unit increase for NDVI_mean, NDVI_sd, per 1km increase for GSC and BSW, per 100 unit increase for IMP and per 10 unit increase for LAN. Cohort-specific and meta-analysis results in table format can be found in **Supplemental Table 10**.

# Supplemental Figure 4 Meta-analysis of single exposure models with asthma incidence in the ambient temperature domain


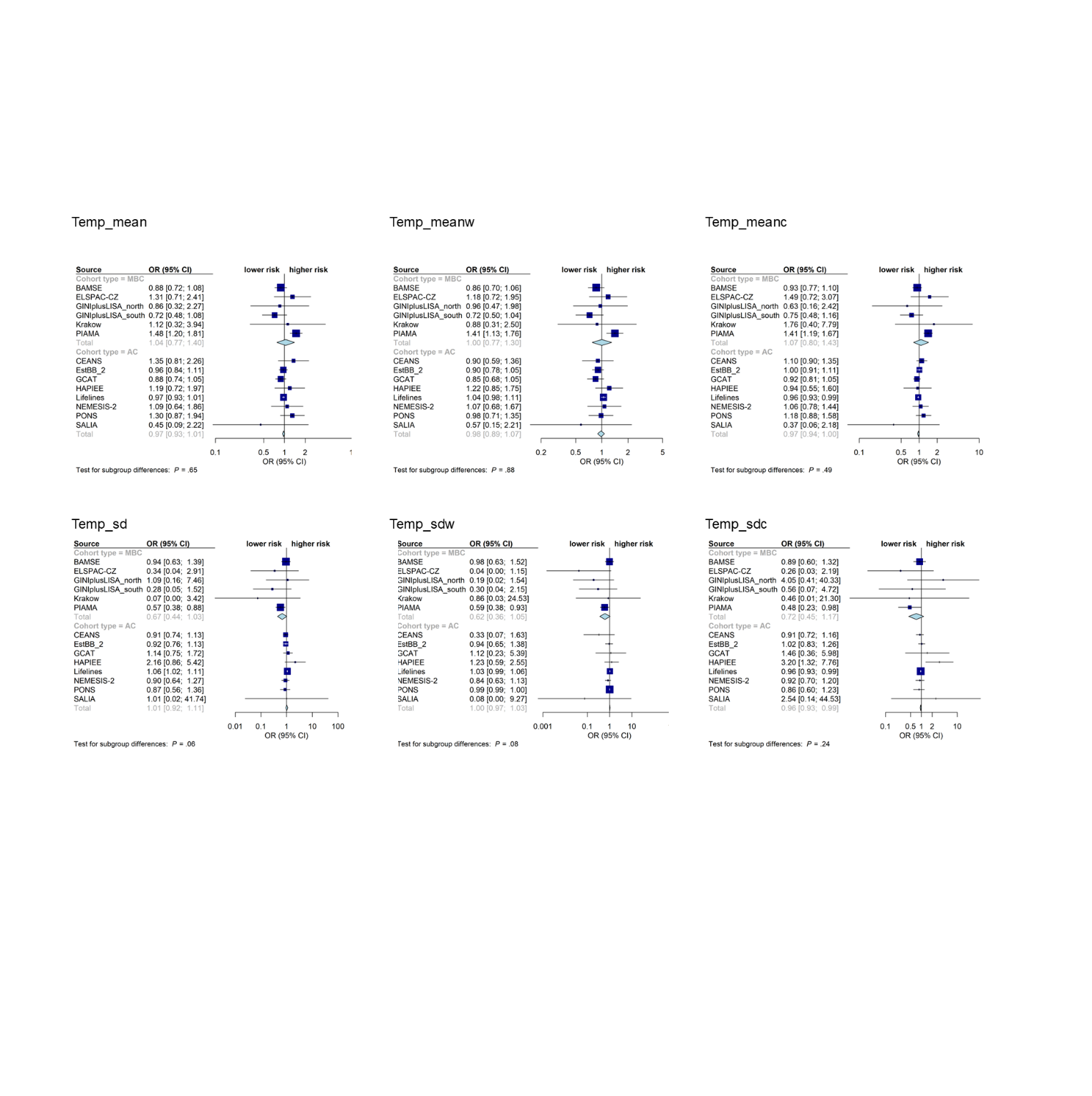


Estimates in the birth cohorts were adjusted depending on the availability in the cohorts for age (dummy variable), sex, parental education, parental asthma/hay fever, breastfeeding, native nationality, day care attendance, older siblings, maternal smoking, environmental tobacco smoking, mould dampness at home, pets, use of gas cooking, active smoking and in the adult cohorts were adjusted depending on the availability in the cohorts for age, sex, smoking status, BMI, marital status, employment, education level, area-level SES.

Odds ratios are presented as per unit increase in mean and standard deviation of the temperatures. Cohort-specific and meta-analysis results in table format can be found in **Supplemental Table 10**.

# Supplemental Figure 5 Cluster-based distribution of air pollution exposures across cohorts


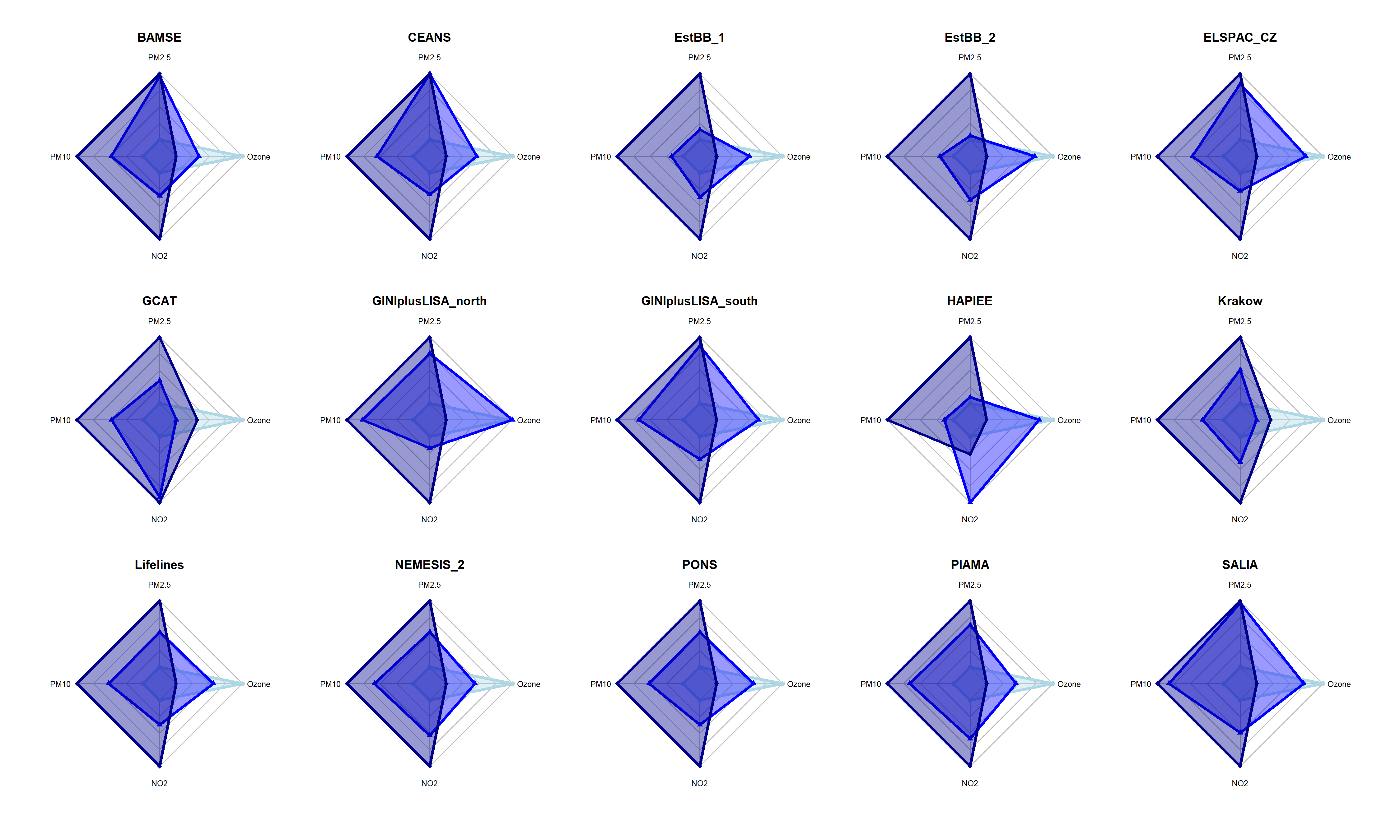


Cohort-specific distribution of air pollution exposure (PM_2.5_, PM_10_, NO_2_ and O_3_) by clusters in the air pollution domain. The axis of the radar plot represents the median levels of specific exposure by clusters. The high PM-NO2 but low O3 clusters were presented in dark blue, the moderate pollution clusters were presented in blue, the clean air with elevated O3 clusters were presented in light blue.

Abbreviation: PM2.5, particulate matter with median aerodynamic diameters <2.5 μm; PM10, particulate matter with median aerodynamic diameters <10 μm; NO2, nitrogen dioxide; O3, Ozone

# Supplemental Figure 6 Cluster-based distribution of built environment exposure across cohorts


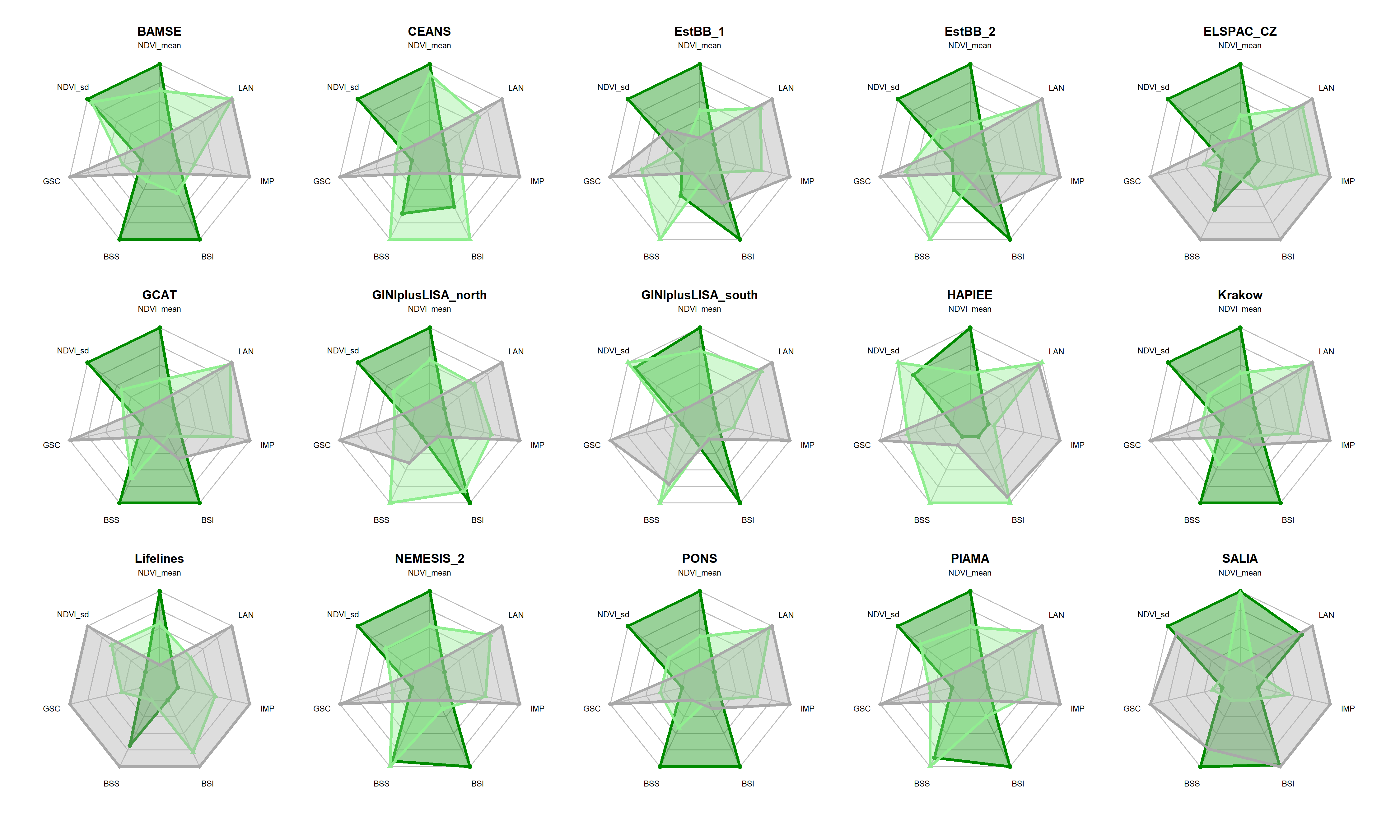


Cohort-specific distributions of exposure (NDVI_mean, NDVI_SD, GSC, BSS, BSI, IMP, LAN) in the built environment domain. The axis of the radar plot represents the median levels of specific exposure by clusters. The clusters with highest greenness, lowest IMP, LAN were presented in dark green, the clusters with moderate greenness, IMP and LAN were presented in light green, the clusters with lowest greenness, highest IMP and LAN were presented in grey.

Abbreviation: BSI_DIS, distance to the nearest inland fresh water; BSS_DIS, distance to the nearest sea; GSC_DIS, distance to the nearest green space according to the Corine database; IMP, imperviousness; LAN, light at night; NDVI_mean, average of the normalized difference vegetation index; NDVI_sd, standard deviation of the normalized difference vegetation index;

# Supplemental Figure 7 Cluster-based distribution of temperature exposure across cohorts


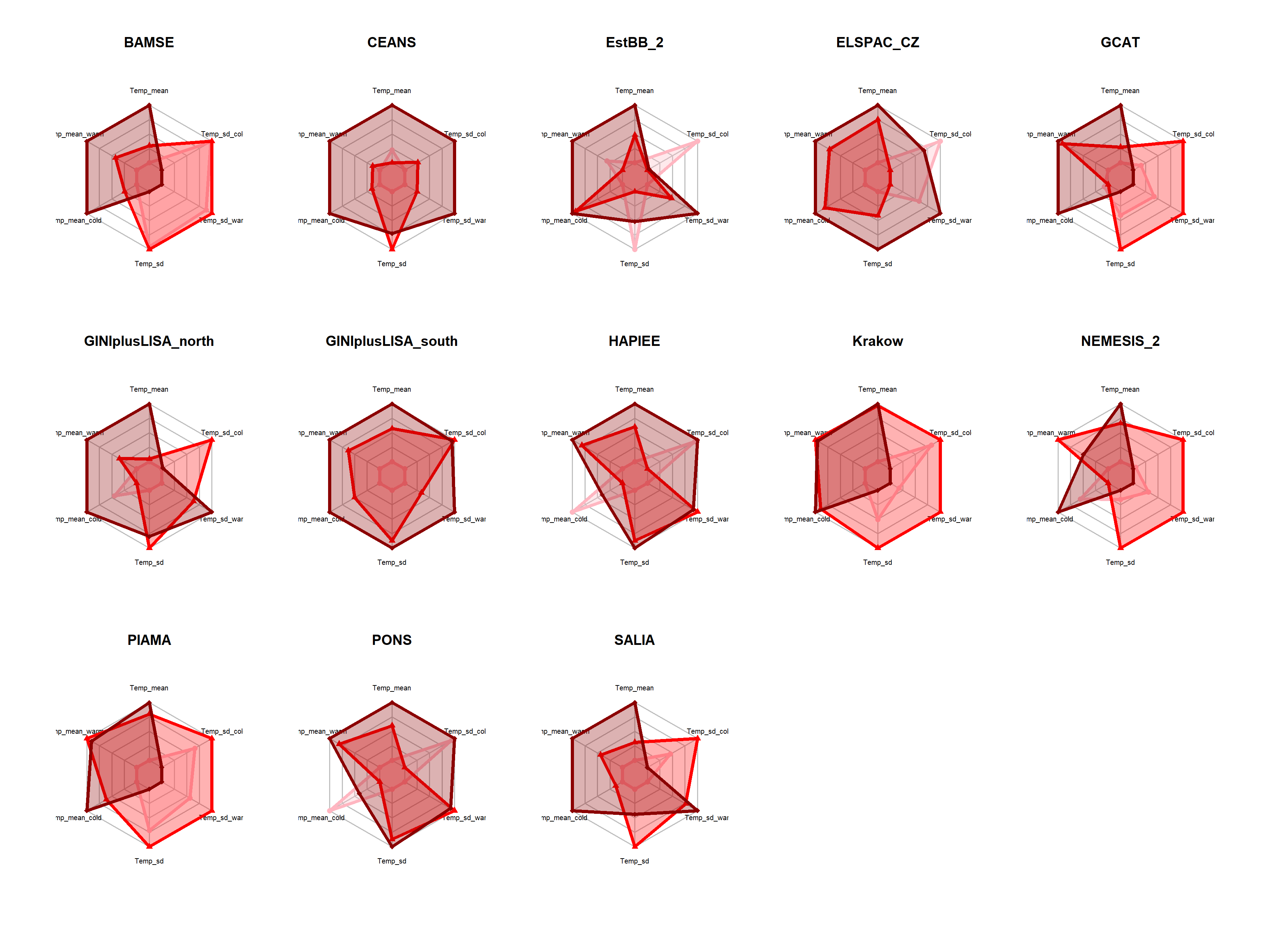


Cohort-specific distributions of exposure (average and standard deviation of the annual/season specific temperature) in the ambient temperature domain. The axis of the radar plot represents the median levels of specific exposure by clusters. The clusters with highest annual mean temperature were presented in dark red; the clusters with moderate mean annual mean temperature were presented in light red, the clusters with lowest annual mean temperature were presented in pink.

Abbreviation: Temp_mean, annual average daily temperature; Temp_mean_cold/warm, average of the daily temperature of the cold/warm season; Temp_SD, standard deviation of the daily temperature of the year; Temp_SD_cold/warm, standard deviation of the daily temperature of the cold/warm season

# Supplemental Figure 8. Distributions of the environmental risk scores across the cohorts


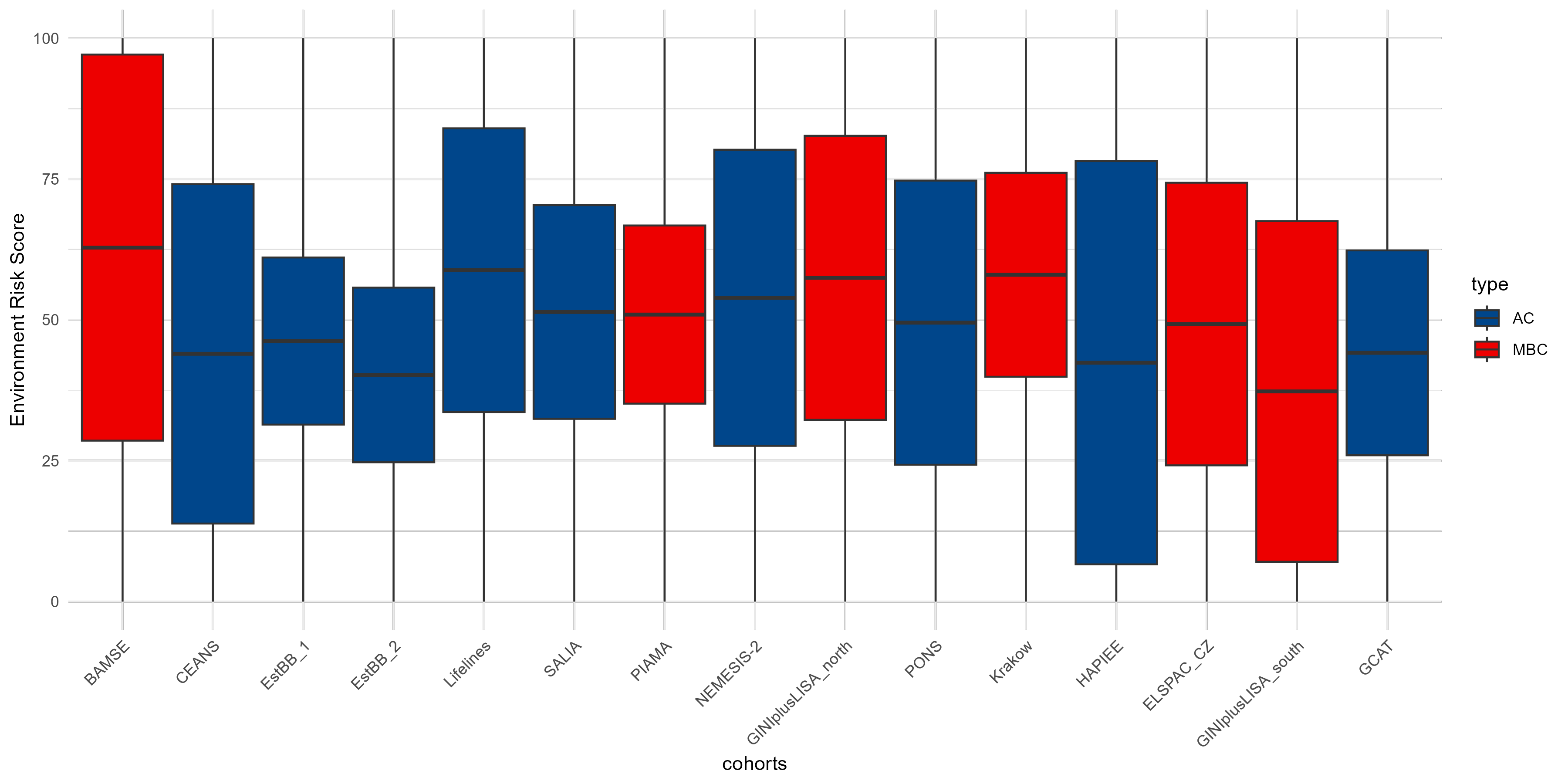


The lower and upper bounds of the box represent the first quartile (Q1) and third quartiles (Q3). The whiskers extend from the Q1 and Q3 to the smallest and largest data points that lie within 1.5 times the interquartile range (IQR) from Q1 and Q3, respectively..


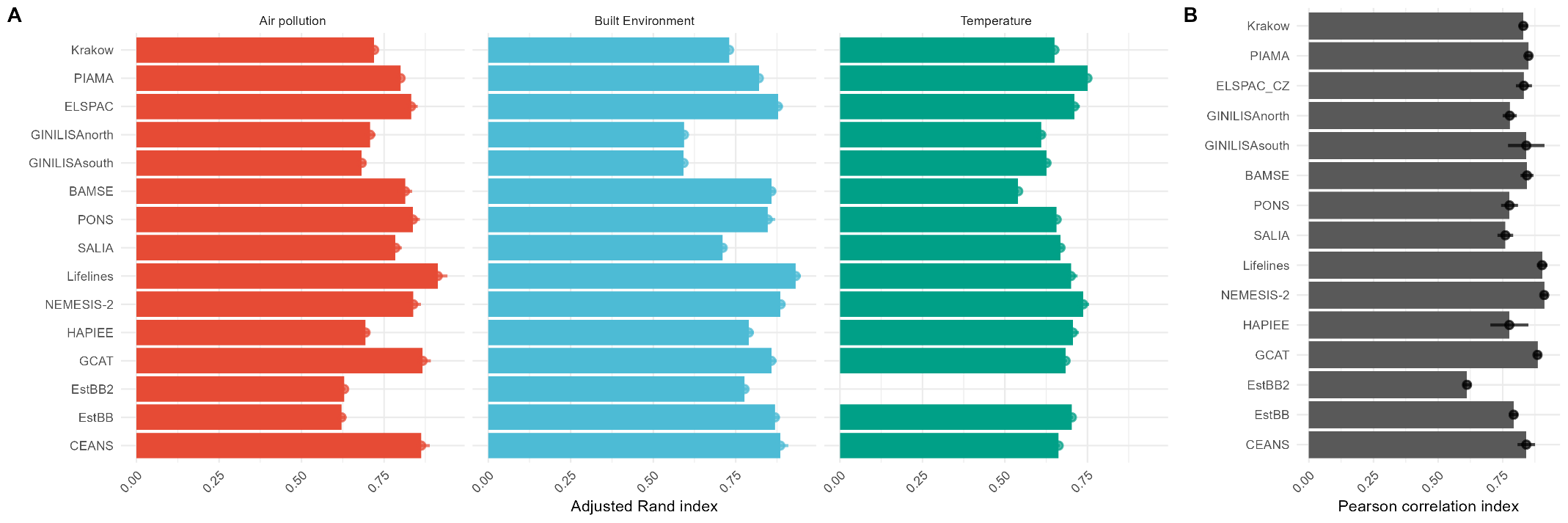


Supplemental Figure 9. Comparison of cluster assignment using adjusted rand index (A) and Pearson’s correlation index for the environment risk score (B) between the cohort-specific and pooled simulated datasets. Larger values of the adjusted rand index indicate a higher similarity of the cluster assignment derived from the simulated single cohorts and pooled datasets. Ambient temperature exposure was not available in the EstBB_2 cohort.

The lower and upper bounds of the box represent the first quartile (Q1) and third quartiles (Q3). The whiskers extend from the Q1 and Q3 to the smallest and largest data points that lie within 1.5 times the interquartile range (IQR) from Q1 and Q3, respectively.

# Supplemental Figure 10 Association between the weighted environmental score with asthma incidence stratified by residential change during the follow-up period.


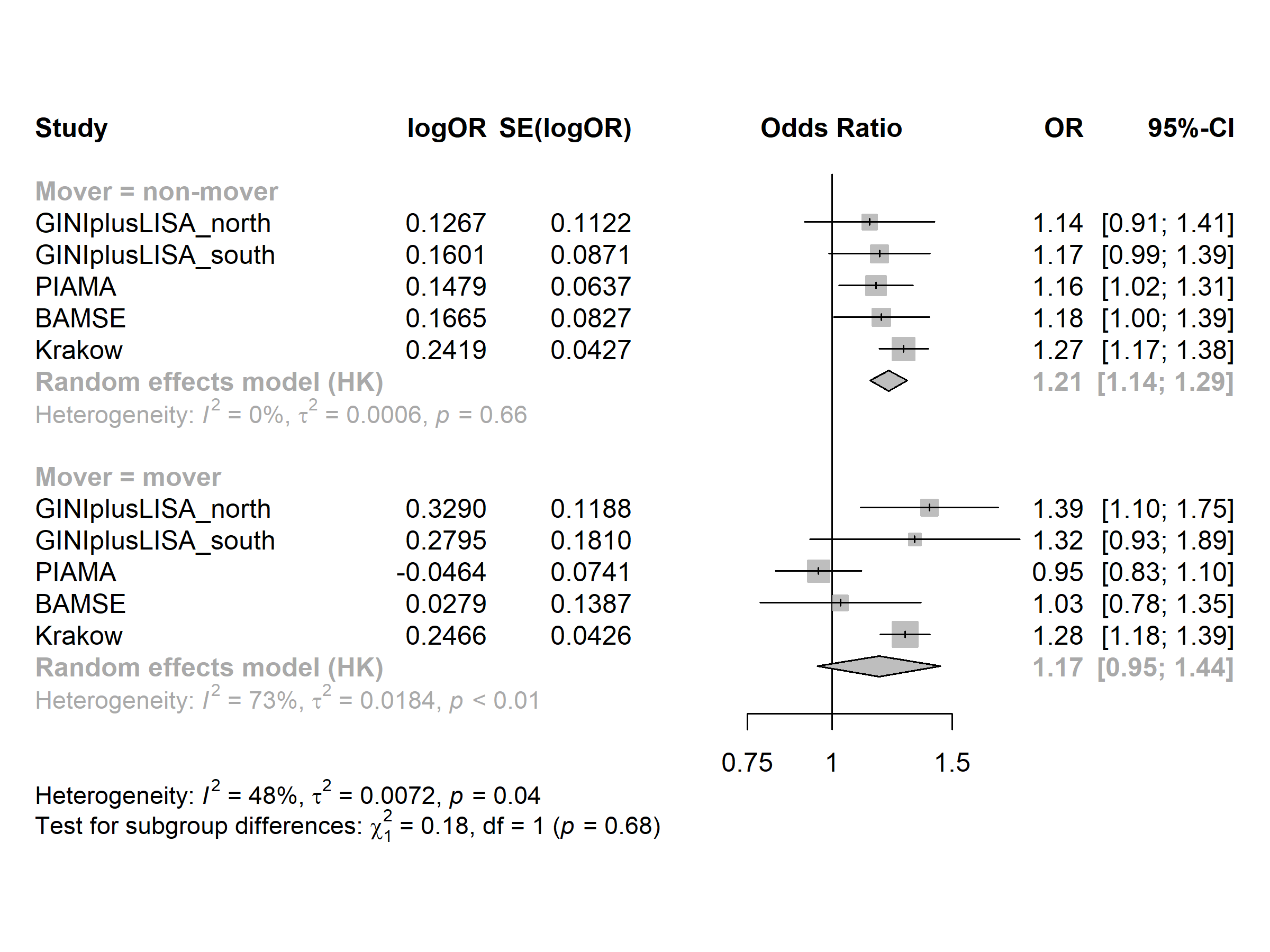


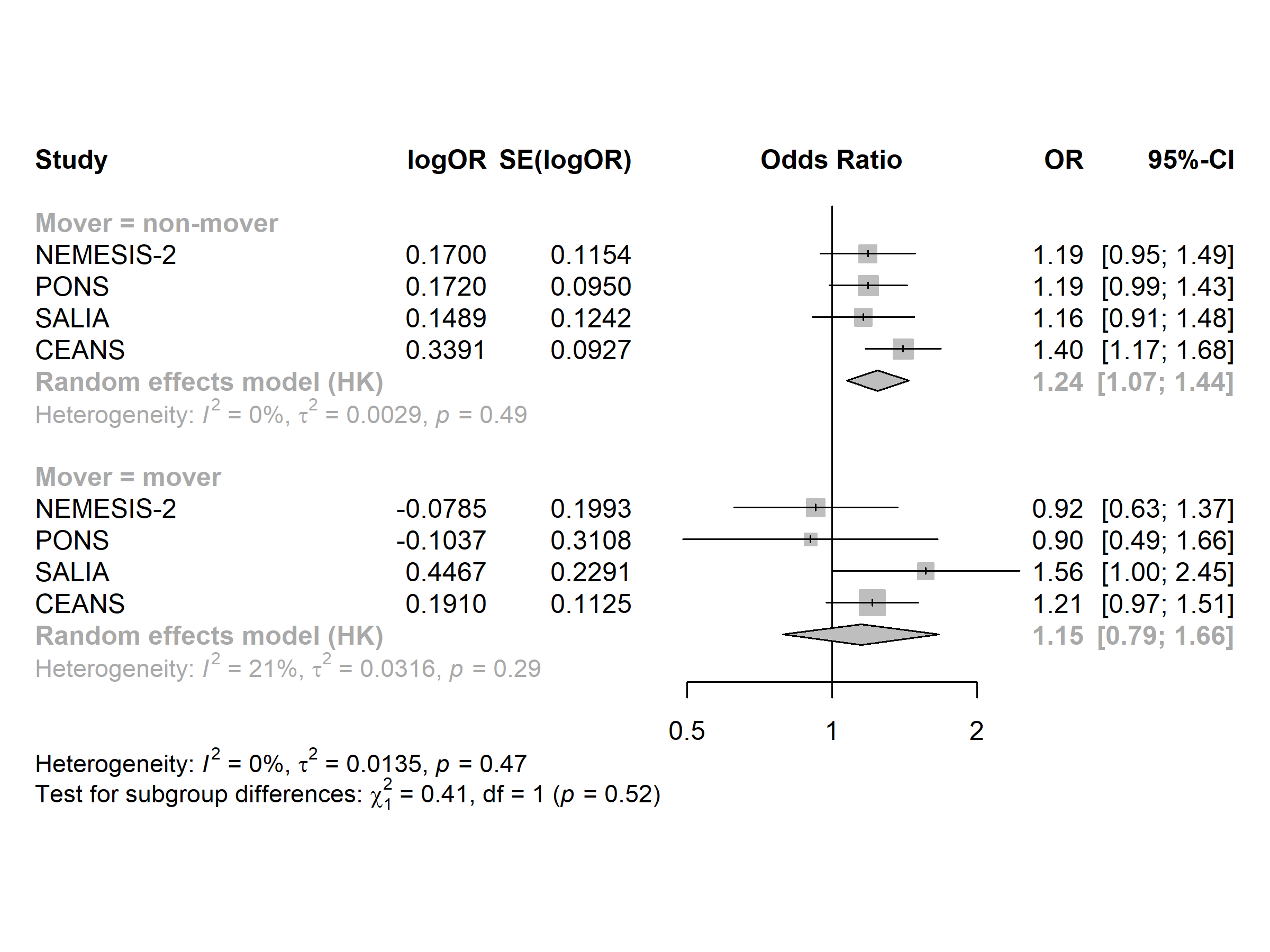


Estimates in the birth cohorts were adjusted depending on the availability in the cohorts for age (dummy variable), sex, parental education, parental asthma/hay fever, breastfeeding, native nationality, day care attendance, older siblings, maternal smoking, environmental tobacco smoking, mould dampness at home, pets, use of gas cooking, active smoking and in the adult cohorts were adjusted depending on the availability in the cohorts for age, sex, smoking status, BMI, marital status, employment, education level, area-level SES.

The OR(95%CI%) was presented as per standard deviation increase. Random-effect meta-analysis with the Hartung-Knapp-Sidik-Jonkman method was used to pool the estimate.

# Supplemental Figure 11 Comparison of the meta-analyzed association between single exposure based on baseline addresses and current addresses in the mature birth cohorts


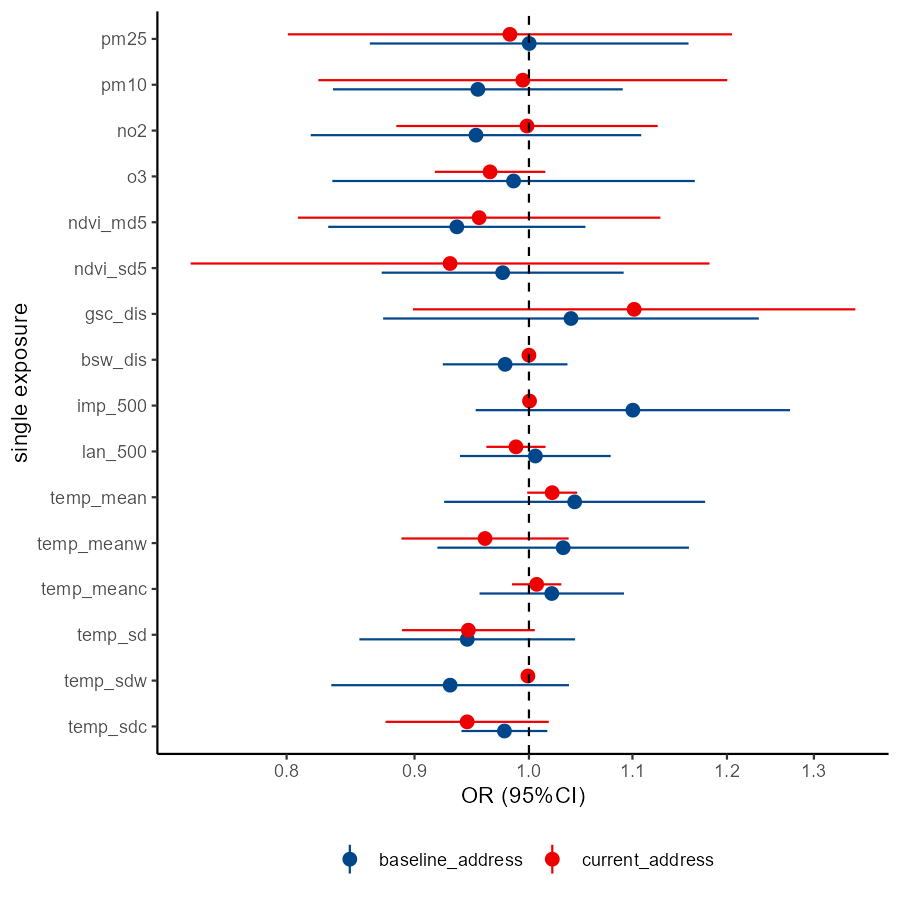


Estimates in the birth cohorts were adjusted depending on the availability in the cohorts for age (dummy variable), sex, parental education, parental asthma/hay fever, breastfeeding, native nationality, day care attendance, older siblings, maternal smoking, environmental tobacco smoking, mould dampness at home, pets, use of gas cooking, active smoking.

Cohort-specific and meta-analysis results in table format can be found in **Supplemental Table 10**.

# Supplemental Figure 12 Sensitivity analysis for the built environmental exposure domain with different buffer sizes


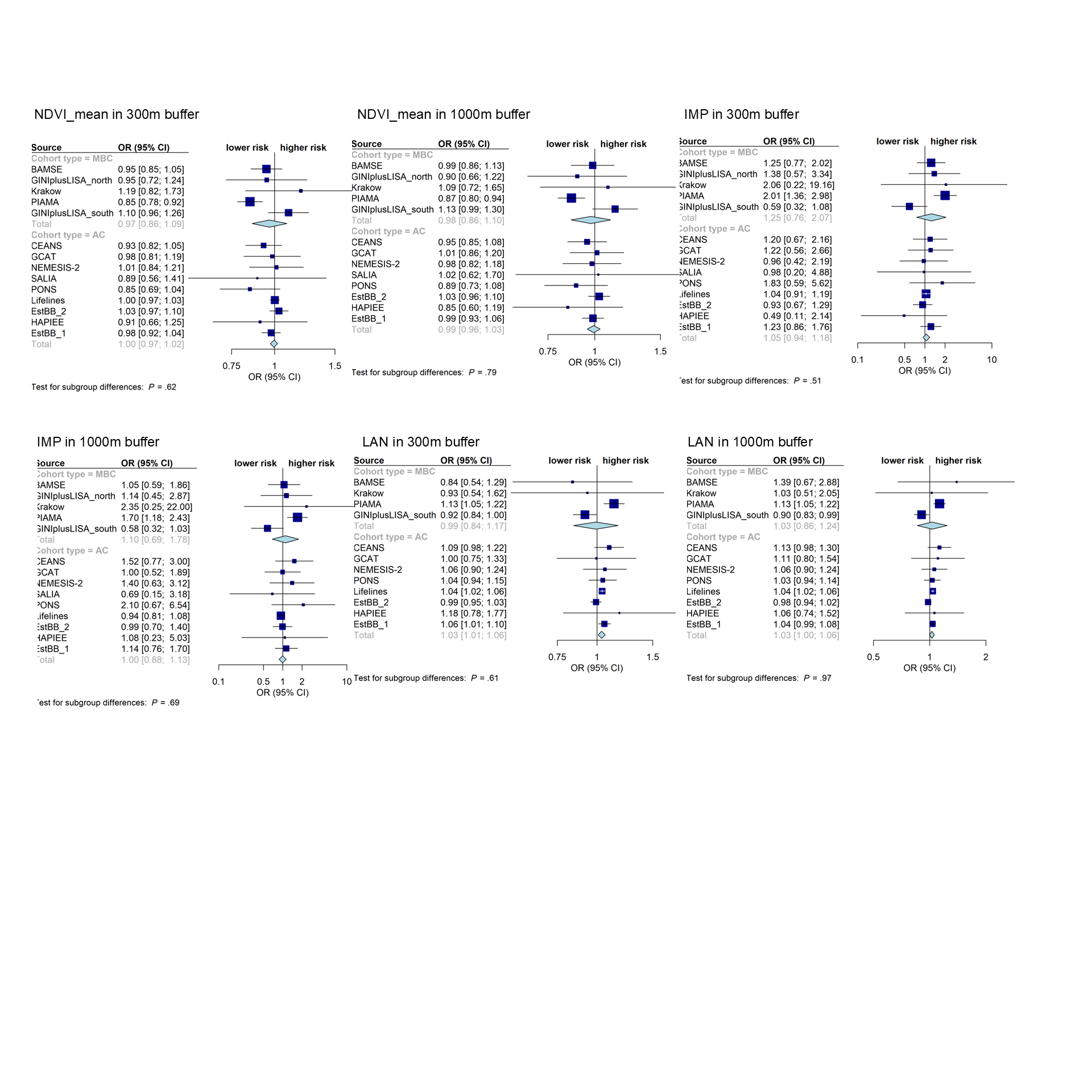


Estimates in the birth cohorts were adjusted depending on the availability in the cohorts for age (dummy variable), sex, parental education, parental asthma/hay fever, breastfeeding, native nationality, day care attendance, older siblings, maternal smoking, environmental tobacco smoking, mould dampness at home, pets, use of gas cooking, active smoking and in the adult cohorts were adjusted depending on the availability in the cohorts for age, sex, smoking status, BMI, marital status, employment, education level, area-level SES.

Odds ratios were presented per 0.1 unit increase for NDVI, er, per 100 unit increase for IMP and per 10 unit increase for LAN.

Cohort-specific and meta-analysis results in table format can be found in **Supplemental Table 10**.

# Supplemental Figure 13 Sensitivity analysis for air pollution exposure based on back-extrapolations


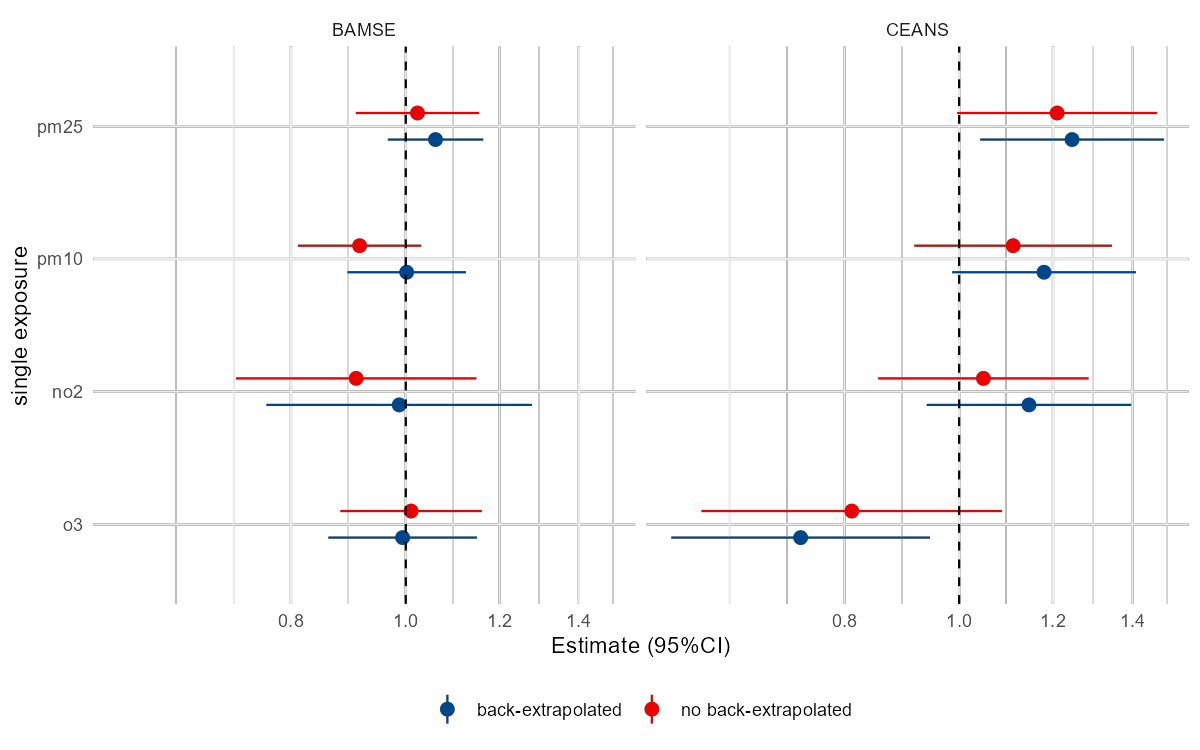


ORs (95%CI) were presented for BAMSE and HRs (95%CI) were presented for CEANS. Estimates in the birth cohorts (BAMSE) were adjusted depending on the availability in the cohorts for age (dummy variable), sex, parental education, parental asthma/hay fever, breastfeeding, native nationality, day care attendance, older siblings, maternal smoking, environmental tobacco smoking, mould dampness at home, pets, use of gas cooking, active smoking and in the adult cohorts (CEANS) were adjusted depending on the availability in the cohorts for age, sex, smoking status, BMI, marital status, employment, education level, area-level SES.

# Supplemental Figure 14 Sensitivity analysis for using follow-up time as time axis in survival analysis in four adult cohorts


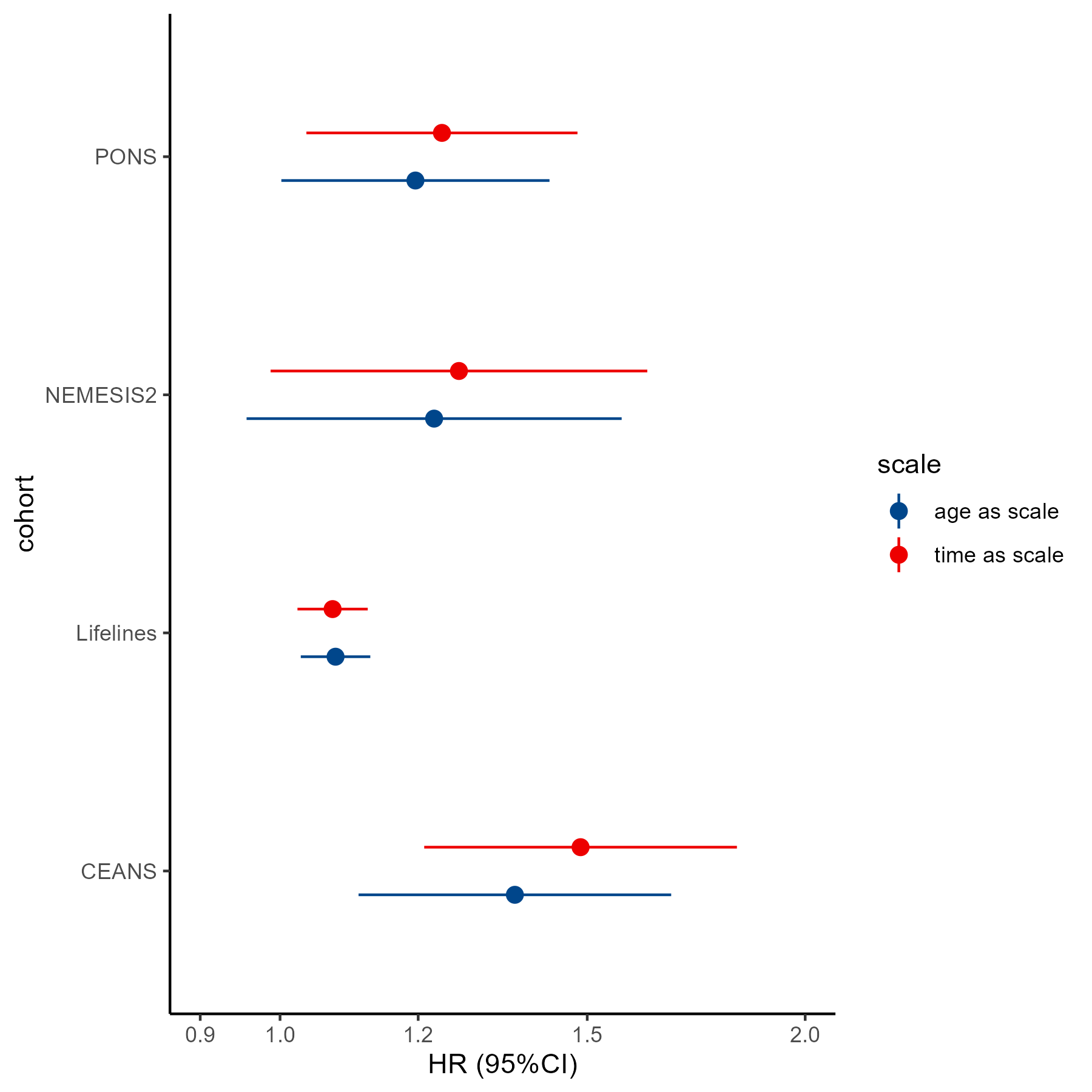


The estimates presented are the environmental risk score per cohort-specific standard deviation increase. Estimates were adjusted for age, sex, smoking status, BMI, marital status, employment, education level, area-level SES. Estimates of the environmental risk score were presented per cohort-specific standard deviation increase.
