# Supplementary material for: External exposome and incident asthma across the life course in 14 European cohorts: a prospective analysis within the EXPANSE project
Source: Lancet Reg Health Eur. 2025 May 15;54:101314. doi: 10.1016/j.lanepe.2025.101314 (PMC12266180; doi:10.1016/j.lanepe.2025.101314)
Supplement: EstBB bannerauthor for pubmed [file mmc3.docx]

| First name | Last name |
| --- | --- |
| Andres | Metspalu |
| Lili | Milani |
| Tõnu | Esko |
| Mait | Metspalu |
